# Quantification of Volatile Organic Compounds (VOCs), Nitrogen Oxides (NO_*x*_), and Ultrafine Particles (UFPs) Emitted by Domestic Air Fryers: A Chamber Study of Indoor Air Quality Impacts

**DOI:** 10.1021/acsestair.5c00363

**Published:** 2026-01-27

**Authors:** Ruijie Tang, Yizhou Su, William Joe F. Acton, Lara K. Dunn, Christian Pfrang

**Affiliations:** † School of Geography, Earth and Environmental Sciences, 1724 University of Birmingham, Edgbaston, B15 2TT Birmingham, U.K.; ‡ Department of Meteorology, University of Reading, Whiteknights, Earley Gate, RG6 6BB Reading, U.K.

**Keywords:** indoor air quality, cooking emissions, exposure assessment, mass balance modeling, air exchange rate, particle size distribution, principal component analysis

## Abstract

Air frying has emerged as a popular low-oil cooking method, yet its impact on indoor air pollutant emissions remains insufficiently understood. In our study, emissions of volatile organic compounds (VOCs), nitrogen oxides (NO_*x*_), and ultrafine particles (UFPs) were measured during the air frying of 12 different dishes within a ca. 0.15 m^3^ Perspex chamber. Pollutant emissions varied significantly depending on the food type, with rates in the ranges of 17.8–184.0 μg min^–1^ for total cooking VOCs, 24.6–37.9 μg min^–1^ for NO_*x*_, and 0.1–17.4 × 10^12^ # min^–1^ for UFPs, primarily due to Maillard reactions and lipid thermal decomposition. While pollutant concentrations and ozone formation potentials were elevated within the chamber, scaling to the volume of a small kitchen indicated substantially lower levels compared to conventional frying methods. Notably, only high-fat foods produced UFP concentrations comparable to those of deep frying. No NO_*x*_ emissions were found during blank (empty appliance) runs, and NO_*x*_ was only detectable while cooking certain types of foods. However, residues accumulating within inaccessible areas of the air fryer following over 70 uses led to increases of 23% in VOC and 236% in UFP concentrations while not cooking food.

## Graphical abstract

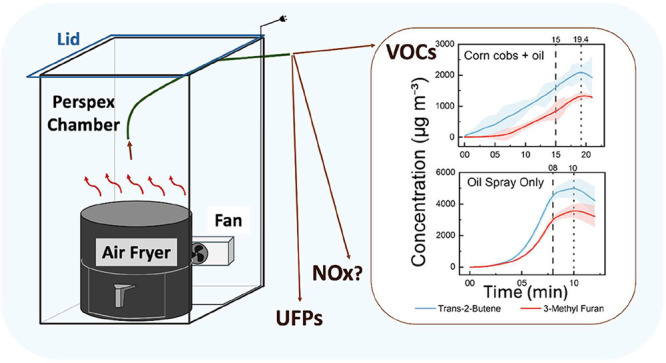

## Introduction

Home cooking releases particles, polycyclic aromatic hydrocarbons (PAHs), and gaseous pollutants, all of which influence indoor air quality.^1–3^ Epidemiological studies have linked exposure to these emissions with various health effects, including cardiovascular diseases and lung cancer.^3–5^ The toxicity profile of cooking emissions depends on multiple factors, including ingredients, cooking temperature, and method.^6–9^ Studies have shown that particle concentrations can vary significantly between cooking methods, indicating that cooking technique is a critical determinant of exposure.^8–11^ High-temperature, oil-based cooking emits aerosols with a higher proportion of fine particles and gaseous compounds compared to water-based cooking.^9,12–14^ Frying produces predominantly ultrafine particles (<100 nm), which are highly efficient at depositing in the respiratory tract.^12,15,16^ Volatile organic compounds (VOCs) are also abundant during frying, often arising from the thermal decomposition of lipids.^8,17–19^ The choice of cooking fuel is another important factor; for instance, gas stoves emit combustion byproducts (e.g., CO and NO_*x*_), in contrast to electric appliances.^1,9^

Air fryers cook food by circulating hot air with little or no added oil, producing foods with significantly lower fat content compared to deep frying.^20,21^ Although this technology is relatively new and detailed emission studies are limited, its popularity is increasing due to growing health awareness around low-oil cooking.^21,22^ Therefore, the widespread use of air fryers warrants investigation into their impact on indoor air quality. It might be expected that using less oil and electric heating would reduce pollutant emissions. However, air fryers operate at high temperatures and employ active convective heat transfer, generating turbulence from internal fans that can enhance the aerosolization of oils and lead to particle formation.^20,21,23^ Some studies indicate that air frying can produce as many particles as conventional cooking methods.^20,21,24^ For example, air frying chicken wings resulted in PM_1__0_ emission factors that were 2–5 times higher than pan-frying, although VOC emission factors were approximately 1.2 times lower than those from sautéing.^21^ This suggests air frying may elevate particulate levels and hence negatively impact indoor air quality.

Few studies have quantitatively characterized the full spectrum of pollutants emitted during air frying. Prior investigations have typically been conducted in home or laboratory kitchens, which involve large volumes and active ventilation, diluting pollutant concentrations.^6,8,9^ Dilution disrupts detecting low-level VOCs or particles and accurately determining emission profiles. Our previous study assessed particulate matter (PM) and VOC emissions from cooking chicken breasts using various methods in an 85 m^3^ research kitchen and determined PM emissions from air frying were comparable to boiling.^11^

To further examine air fryer emissions, we conducted the present study in a controlled chamber equipped with a fan to ensure a well-mixed air. This tightly controlled environment allows emitted pollutants to accumulate to detectable levels, overcoming limitations associated with dilution.^11^ By isolating the cooking process, we could accurately quantify emission rates and capture the chemical compositions before they were dispersed by ventilation. Representative foods were selected for experimentation, and the study focused on assessing emission profiles, including peak concentrations, emission rates, and factors for VOCs, NO_*x*_, and particles, and estimations of ozone formation potentials (OFP). This study provides critical insights into the impact of air frying on indoor air quality.

## 2 Materials and Methods

### 2.1 Design of Chamber and Air Frying Procedure

#### 2.1.1 Chamber and Air Fryer Design

Cooking experiments were conducted in a custom Perspex chamber (45 × 45 × 75 cm; 151.9 L) equipped with a 12 V DC brushless fan, airtight seals, and a removable lid with a 1 cm gap along one edge to admit sampling tubing and maintain pressure balance as the air fryer elevated the temperature and humidity. The inlet port was placed centrally, about 25 cm below the lid. Before each run, the chamber was inspected and cleaned to minimize residual particles or VOCs. The chamber was situated in a ventilated laboratory at the University of Birmingham.

A 4.7 L COSORI air fryer (27.2 × 27.5 × 30.3 cm; model CAF-L501-KUK) was centered in the chamber with its top heating element and rear outlet aligned to the chamber fan. The test unit was a basket-style consumer air fryer employing an overhead electric heating coil and convection fan with top/rear exhaust vents, representative of widely sold models in this category. The outlet, located about 18 cm above the chamber floor, exhausts heated air by diffusion; we set the fan at the same height to optimize mixing. Detailed dimensions and vendor information on the chamber and air fryer are provided in SI Text S1, with the protocols of cooking and cleaning stated in the SI Text S2. Figure 1 shows the overall experimental setup including chamber layout and instrumentation.

**Figure 1:**
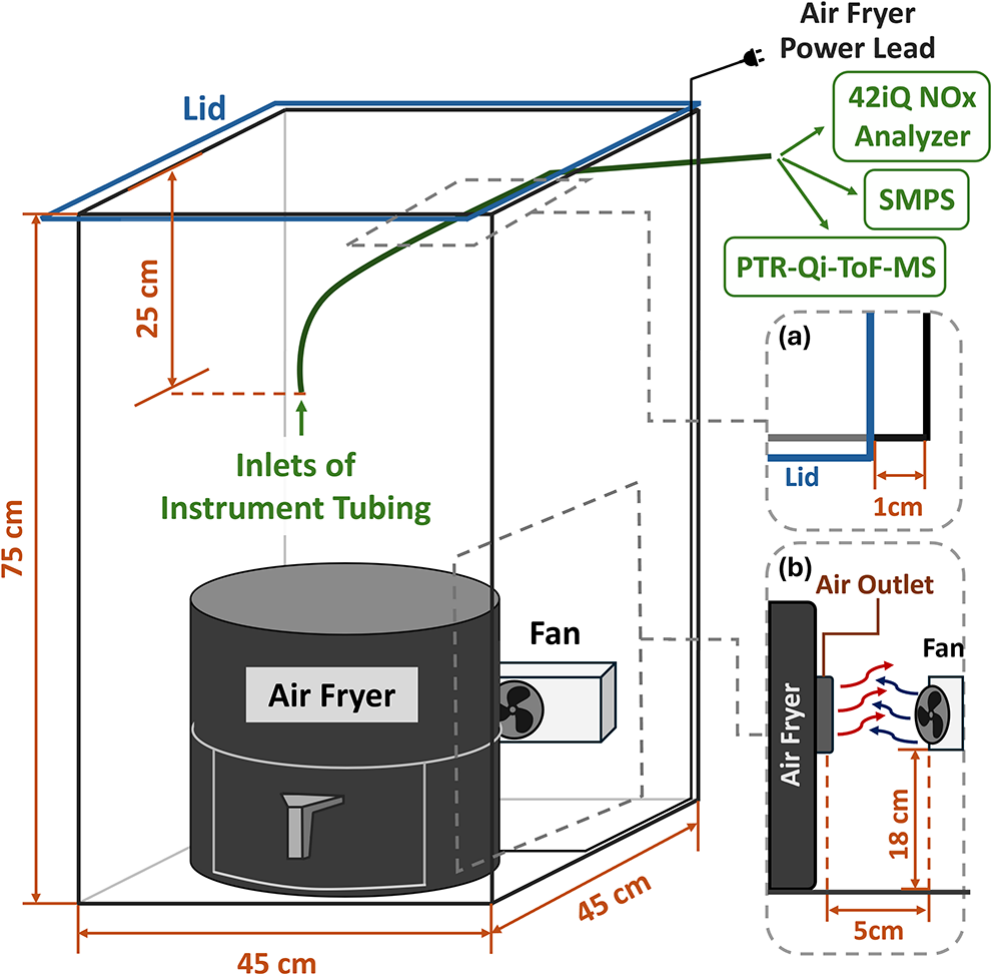
Layout of the chamber design: positions of the air fryer, fan, and instrument-tubing inlets. Insets: (a) 1 cm gap by the lid for tubing access; (b) air-fryer outlet and fan locations.

#### 2.1.2 Cooking Procedures

Twelve dishes commonly chosen for frying or oven-cooking, three frozen fried foods, five low-fat foods, and four high-fat foods, were tested for at least three times (Table 1), and their nutrient composition (fat, protein, carbohydrate) were reported in SI Table S1. Before each run, samples were weighed to ±0.1 g; low-fat items received a 0.86 g spray of rapeseed oil to ensure browning. At the start of each experimental day, the air fryer was preheated empty at 205 °C for 4 min, followed with a recording of 9 min blank run at 175 °C for establishing baseline pollutant levels. The 9 min runs with 0.86 g of oil spray only were conducted as well.

**Table 1:** Summary of Dishes, Abbreviations, Food Types, Weights, and Cooking Durations (*t*_cook_)

dishes (number of replicates)^b^	abbreviation of dishes	food type	weight (mean ± standard deviation, g)	*t*_cook_ (min)
background (empty) (5)	BG	N/A	0	9
five sprays of rapeseed oil applications (5)	oil only	N/A	0.86 ± 0.03	9
frozen fried chicken breast (5)	frozen FCB	frozen fried food	170^a^	18
frozen smiley hash brown (6)	frozen SHB	frozen fried food	147.3 ± 0.6	12
frozen onion rings (5)	frozen OR	frozen fried food	150.7 ± 4.2	14
frozen broccoli and cauliflower with oil sprays (5)	frozen B&C + oil	oiled low-fat food	302.0 ± 9.5	9
fresh chicken breast slices with oil sprays	fresh CB + oil	oiled low-fat food	151.3 ± 3.1	14
courgette slices with oil sprays (5)	courgette + oil	oiled low-fat food	151.3 ± 7.6	12
corn on the cobs with oil sprays (5)	corn cobs + oil	oiled low-fat food	383.3 ± 12.1	15
mushrooms with oil sprays (5)	mushroom + oil	oiled low-fat food	221 ± 12.8	9
vegetarian sausages (5)	V sausages	high-fat food	182.3 ± 0.6	15
pork sausages (5)	P sausages	high-fat food	228.3 ± 0.6	22
unsmoked bacon (5)	U bacon	high-fat food	61^a^	9
smoked bacon (6)	S bacon	high-fat food	61^a^	9

a The items are standard commercial products of fixed manufacturers’ weight, measured in whole grams only.

b Numbers in parentheses indicate total runs performed per dish. The three final replicates per dish were used for statistics and figures.

Dish cooking runs were carried out at 175 °C using manufacturer’s recommended durations or times optimized in preliminary trials. Preweighed food was loaded and cooked for the prescribed period, with no manual intervention needed during cooking because of the built-in timer of the air fryer, which could stop heating automatically. After cooking, the lid remained closed until the pollutant time series reached a clear maximum and began to decline, and then measurements were continued for a 10 min decay period; then, the air fryer was taken out to leave the chamber ventilating for 15 min before the next trial. No further preheating was required between successive runs. Detailed descriptions of the cooking and cleaning protocols are provided in SI (Text S2).

### 2.2 Monitoring of VOC, NOx, Particles, and Environmental Conditions

#### 2.2.1 VOCs and Cooking-Related VOCs

VOC mixing ratios (in parts per billion, ppb) were measured by a proton-transfer-reaction quadrupole-ion-time-of-flight mass spectrometer (PTR-Qi-ToF-MS, Ionicon Analytik GmbH, Innsbruck, Austria) within the chamber. This instrument has been described in detail previously.^25,26^ The inlet flow was set to 40 mL min^–1^, and the drift tube was maintained at a pressure of 3.8 mbar, a voltage of 850 V, and a temperature of 79.9 °C. The corresponding reduced electric field (*E*/*N*), where *E* denotes the electric field strength and *N* represents the gas molecule number density, was 120 Td (i.e., 1.2 × 10^–15^ V cm^2^ molecule^–1^). Data were acquired at a one-second time resolution and analyzed using PTR-MS Viewer version 3.4.^27,28^ The instrument was calibrated using a multicomponent gas standard (Apel-Riemer Environmental, Miami) (compound-specific calibrations ∼±20%, default sensitivities ∼±30–40%). VOC species were identified by comparing the measured mass-to-charge (*m*/*z*) ratios of ionized compounds against established reference libraries and literature on cooking-related emissions.^11,29^

Cooking-related VOCs (CVOC) were identified by principal component analysis (PCA) in IBM SPSS Statistics Version 30.0 to cluster VOC species associated with cooking. We applied Varimax rotation to the time series of 326 *m*/*z* signals, selecting components via the Kaiser criterion (eigenvalues >1), scree-plot inspection, and communalities. Only components with loadings >0.6 whose temporal profiles corresponded clearly to air-fryer on/off cycles were retained.^30–32^ Background mixing ratios before cooking were subtracted before analysis. Finally, linear regression against the time series of the frying state confirmed the selected CVOCs. Detailed PCA workflow, selection criteria, and quantification are mentioned in SI (Text S4).

#### 2.2.2 NO_*x*_

NO_*x*_ measurements were performed using a Thermo Fisher Scientific Model 42i (NO-NO_2_-NOx) chemiluminescence Analyzer (TEI; Franklin, MA, USA). The analyzer measures ambient nitric oxide (NO) level, with a flow rate of 0.6 L min^–1^, directly via its reaction with ozone, while NO_2_ is determined indirectly after conversion to NO in a heated molybdenum converter which operates at approximately 325 °C at a time resolution of 1 min.^33^ The analyzer was calibrated by colocation with a reference NO_*x*_ instrument at the Birmingham Air Quality Supersite (BAQS) and undergoes routine checks, ensuring data accuracy and comparability to ambient standards. Chemiluminescent NO_*x*_ measurements can be biased by humidity and alkene interference, and the molybdenum converter can reduce some NO_*y*_ (e.g., HNO_3_, peroxyacetyl nitrate (PANs), alkyl nitrates).^34^ Accordingly, reported NO_2_ should be interpreted as an upper bound with an uncertainty on the order of ∼10–30% (and up to ∼50% under NO_*y*_-rich/photochemically aged conditions).^34–36^ We did not apply a post hoc correction (converter-specific quantification was unavailable) and instead carry this uncertainty into the interpretation of NO_*x*_ results.

#### 2.2.3 Particles

During the cooking experiments, particles in the size range of 14.6–637.8 nm were measured using a Scanning Mobility Particle Sizer (SMPS, TSI Inc., USA). The system comprised a Model 3080 electrostatic classifier equipped with a Model 3081 long Differential Mobility Analyzer (DMA), coupled with a Model 3075 Condensation Particle Counter (CPC). In this configuration, the DMA operated with dry sheath/inline drying and separated particles by electrical mobility for size-resolved measurement, while the CPC counted them, providing simultaneous concentration and size-distribution data throughout each cooking run.^15,16^ Postcook decay fits yielded size-resolved total removal λ, which inherently includes ventilation, wall/surface deposition, and any coagulation, and was used solely to compute emission rates (mass balance) and not to adjust concentrations. Diffusional line losses for the short conductive tubing were estimated to be a few percent and were not corrected.

#### 2.2.4 Environmental Monitoring and Air Exchange Rate

The mixing ratios of CO_2_, relative humidity (RH, %), and temperature (°C) were monitored using Aranet4 Home monitors. One monitor was placed at the top of the air fryer, while the other was located outside the chamber to monitor the ambient laboratory conditions. Based on the manufacturer’s specifications, the monitors measure CO_2_ from 0–9999 ppm (1 ppm resolution; ±30 ppm +3% for 0–5000 ppm), temperature from 0–50 °C (0.1 °C resolution; ±0.3 °C), and RH from 0–85% (1% resolution; ±3%). Data were recorded via the Aranet4 app and exported for decay analysis.

The air exchange rate (AER, also known as ‘air change rate (ACR)’ and ‘air change per hour (ACH)’) of the chamber was determined using the CO_2_ decay method.^37^ All instruments were kept operating under their standard conditions during the measurement. This included the PTR-Qi-ToF-MS, Model 42i Analyzer, and SMPS, all of which draw air at a fixed flow rate during operation, as well as the air fryer, which was also kept running for the CO_2_ assessment at 175 °C with a bowl of water in the tray to replicate experimental conditions. CO_2_ was injected into the chamber to ca. 3000 ppm, after which the chamber was sealed without tubing of the instrument placed in the chamber to allow thorough mixing facilitated by the mixing fan. After the CO_2_ concentration stabilized, the instrument tubing was reinserted and the system was then left under the same operational settings as during the experimental campaign, and the decay of CO_2_ concentration was monitored as it returned exponentially to background levels. The AER was subsequently calculated using eq 1.^37^

$$a=\frac{\ln C_{\left(t\right)}-\ln C_{0}}{t_{i}}$$ 1

where *a* is the AER (h^–1^); *C*_0_ is the initial CO_2_ mixing ratio (ppm) in chamber; *C*_(*t*)_ is the final CO_2_ mixing ratio (ppm) in chamber; and *t*_*i*_ is the time spent for ventilation (h). In our study, the AER in the chamber was 6.16 ± 0.09 and 1.65 ± 0.05 h^–1^ when the air fryer was on and off, respectively, while previous studies indicated the AER at residences ranged 0.05–4.87 h^–1^.^38,39^

### 2.3 Emission Calculations

After obtaining the raw data, quantitative emission metrics were calculated to characterize the pollution from each cooking event. In particular, we determined the Emission Rate (ER) for each pollutant and the Emission Factor (EF) for VOCs, which indicated the amount of pollutant emitted over a given time period and per unit mass of food, respectively.^40,41^ All calculations assumed that the chamber air was well mixed and employed a single-zone mass balance model, treating the chamber as an approximative sealed space with a slow air exchange.

#### 2.3.1 Emission Rate (ER) for Gaseous Pollutants

VOC data from the PTR-Qi-ToF-MS, originally recorded at 1 s intervals, were averaged over 15 s to reduce noise. The change in concentration, Δ*C*, used in the emission rate equation was computed from these 15 s averages. Additionally, the measured mixing ratios (in ppb) of VOC and NOx, output directly from the instrument, were converted to mass concentration (μg m^–3^), assuming the temperature at 40 °C and pressure at 1006 hPa, based on the observed chamber conditions (temperature 32–47 °C and pressure 1004–1008 hPa) during cooking processes.

The real-time concentration of gaseous pollutants was derived from the mass balance equation by eq 2, which was rearranged to eq 3 for yielding the emission rate.^42,43^

$$C_{\mathrm{in},g}(t)=\left[C_{\mathrm{in},g}(t_{0})-\frac{S_{g}}{aV}\right]e^{-a\Delta t}+\frac{S_{g}}{aV}$$ 2

$$S_{g}=-aV\frac{\Delta C}{1-e^{-a\Delta t}}$$ 3

where *S*_g_ was the gas emission rate (μg s^–1^, then converted to μg min^–1^); *a* was the air exchange rate (s^–1^); *V* was the volume of the chamber (m^3^); Δ*t* was 15 s; and Δ*C* was the change of the 15 s averaged concentrations (μg m^–3^) between successive time points.

For NO_*x*_, the same equations were applied, but because NO_*x*_ data were recorded at 1 min intervals, the *S*_g_ was expressed as the ER (μg min^–1^); Δ*t* was 1 min; and Δ*C* was the change of concentrations between successive 1 min readings.

#### 2.3.2 Emission Rate (ER) for Particles

Particle emission rates were calculated similarly, with additional considerations of the particle loss processes in the chamber. Assuming that the chamber air was well mixed and in steady state, eq 4 stated the calculation of particle emission rate during the cooking process by using a material-balance approach^42^

$$\frac{dC_{\mathrm{in},p}(t)}{dt}=aPC_{\mathrm{out}}-\lambda C_{\mathrm{in},p}(t)+\frac{S_{p}}{V}$$ 4

where *C*_in,p_(*t*) was the real-time chamber particle concentration (# m^–3^); *a* indicated the air exchange rate (min^–1^); *P* represented the penetration factor for particles entering the chamber; *C*_out_ was the particle concentration outside of the chamber (lab particle concentration, # m^–3^); λ referred to the total particle removal rate within the chamber (accounting for coagulation, deposition, and air exchange) as determined from the decay of particle concentration following the peak of each air frying event; *S*_p_ indicated the particle emission rate (# min^–1^); and *V* was the volume of the chamber (m^3^).^13,42^

Under the assumption that the particle concentration was at steady state prior to cooking (i.e., *C*_in,p_(*t*_0_) = *aPC*_out_/λ), due to no other activities before cooking for a period of at least 10 min, eq 4 could be rearranged to eq 5:

$$S_{p}=\lambda V\frac{\Delta C_{\mathrm{in},p}(t)}{1-e^{-\lambda \Delta t}}$$ 5

where Δ*C*_in,p_(*t*) represented the change in particle concentrations between successive measurements taken every 135 s during the SMPS cycles. Detailed steps of equation conversion can be found in our previous studies.^10,11^

For context, we estimate kitchen-scale UFP peaks for a 15 m^3^ small kitchen scenario using a well-mixed mass balance with removal by AER and deposition (*D*_p_) based on the ER assessed in the chamber. We adopt median values of AER = 1 h^–1^ and *D*_p_ = h^–1^ (thus λ = 2 h^–1^). Full equations are provided in the SI (Text S3). We adopt 15 m^3^ as an intentionally conservative small-kitchen example, consistent with EU consumer exposure defaults used in scenario modeling.^44^ Typical kitchens reported in measurement/modeling studies are larger (∼25–40 m^3^), so room-average concentrations in such spaces would be lower for the same emissions.^45,46^

#### 2.3.3 Emission Factor (EF) for VOCs

The emission factor (EF) for VOCs was calculated as

$$\mathrm{EF}=\frac{S_{g}\times T}{W}$$ 6

where *S*_g_ was the VOC emission rate (μg min^–1^); *T* was the total duration from the start of air fryer operation to the peak of VOC concentration (min); and *W* stood for the weight of the food (g).^40^ EF values were reported in μg VOC per g of food. This normalization allowed for direct comparison of pollutant emissions across dishes with different food masses.^19,40^

### 2.4 Ozone Formation Potential (OFP)

The ozone formation potential (OFP) is calculated to assess the potential impact of the emitted VOCs on ozone formation for each cooking experiment. In general, the formation potential of secondary products, such as ozone, aerosols, and PAN, generated by photochemical reactions of VOC species can be estimated by using the incremental reactivity (IR) method. This approach quantifies the change in ozone concentration resulting from small changes in a specific VOC, in the presence of NO_*x*_ and sunlight, thereby representing the ratio of the ozone change to the change in VOC concentration.^47^ The maximum increment reactivity (MIR) developed by Carter^29^ is determined by controlling the NOx concentration for VOCs in various gas mixtures and is considered as the most recognized method for OFP assessment.^18,48^

The OFP of each VOC species was calculated by multiplying the mass concentration of that species by its MIR value. The total OFP for a cooking event was then obtained by summing the contributions from all VOC species, expressed in eq 7:

$$\mathrm{OFP}=\sum_{i=1}^{n}({\mathrm{VOC}}_{i}\times {\mathrm{MIR}}_{i})$$ 7

where VOC_*i*_ represented the mass concentration (μg m^–3^) of a specific VOC species; MIR_*i*_ stated the MIR value (g O_3_ per g VOC,^29^ mathematically equals to μg O_3_ per μg VOC) of this specific VOC species. This calculation indicated the contribution to potential indoor ozone formation of the VOCs emitted by each air-fried dish.

### 2.5 Statistical Analysis and Plotting

Group differences among food types were evaluated with Kruskal–Wallis tests and Dunn–Holm posthoc comparisons. Two-group contrasts used Mann–Whitney *U* and associations with weight loss used Spearman’s ρ. Data analysis was conducted on Microsoft Excel, IBM SPSS (v30.0), and plotting was performed using Origin 2025.

## 3 Results and Discussion

### 3.1 Volatile Organic Compounds (VOCs)

#### 3.1.1 Chemical Composition and Ozone Formation Potential (OFP)

Air frying different foods produces a complex mixture of VOCs, with both the composition and the ozone formation potential (OFP) varying widely by dish. The peak concentrations of total cooking-related VOCs (CVOCs) range from 1.13 to 16.99 mg m^–3^, while the corresponding OFP varies from 5.38 to 122.96 mg m^–3^, depending on ingredient composition. The assessment of background VOC emissions from operating the air fryer with an empty tray revealed only low levels of propene, acetaldehyde, and acetic acid, with a mean total CVOC concentration of 82.8 μg m^–3^. This background emission is attributed to the residuals in those parts of the air fryer that are not accessible to be routinely cleaned. Due to its insignificant amount in comparison to the total CVOCs, and taking into account that these are inevitable emissions from the air fryer operation, the detected baseline emissions are included within further measurement results of cooking trials, as they also reflect real-use exposure.

Figure 2 illustrates the peak CVOC concentrations and the OFP for each dish. In general, the oiled low-fat and high-fat foods tend to emit higher levels of CVOCs, resulting in greater OFP in comparison to frozen fried foods (Kruskal–Wallis: CVOC peaks and OFP, *p* < 0.05; post hoc Dunn–Holm: high-fat > frozen fried, *p* < 0.05; oiled low-fat vs frozen fried, not significant (NS)), with the exception of onion rings and oiled corn on cob. See SI Table S1 for a nutrient context. As the measurements were conducted in a small, enclosed chamber, the recorded concentrations are elevated, which therefore cannot be directly comparable to real-world conditions. Assuming the same mass of pollutants would be emitted into a well-mixed 15 m^3^ kitchen,^43^ the peak concentrations would be approximately 0.011–0.17 mg m^–3^, and the OFP would range from 0.054 to 1.24 mg m^–3^. For comparison with guidelines and the literature, we additionally report the sum of all PTR-MS-measured VOCs (background-subtracted): chamber concentrations ranged from 1.66 to 23.60 mg m^–3^, corresponding to 0.017 to 0.24 mg m^–3^ when scaled to a 15 m^3^ kitchen. According to UK Government Building Regulations 2010,^49^ the peak levels of summed PTR-MS-measured VOCs from air frying in the estimated real-world kitchen would always remain below the total volatile organic compound (TVOC) guidance value of 0.3 mg m^–3^ for an 8 h average exposure time. This estimated real-world concentration also matches results of our previous study, which monitored the total VOC peak concentrations from cooking oily fresh chicken in the same air fryer to range from 0.019 to 0.095 mg m^–3^.^11^ In addition, these values are considerably lower than those reported for VOC emissions and corresponding OFP from other cooking methods, such as deep-frying and stir-frying, using gas stoves or electric hobs, which typically yield total VOC concentrations of 0.26–2.7 mg m^–3^.^48,50^ This suggests that air fryers can offer a cleaner alternative to deep frying in terms of VOC emissions.

**Figure 2:**
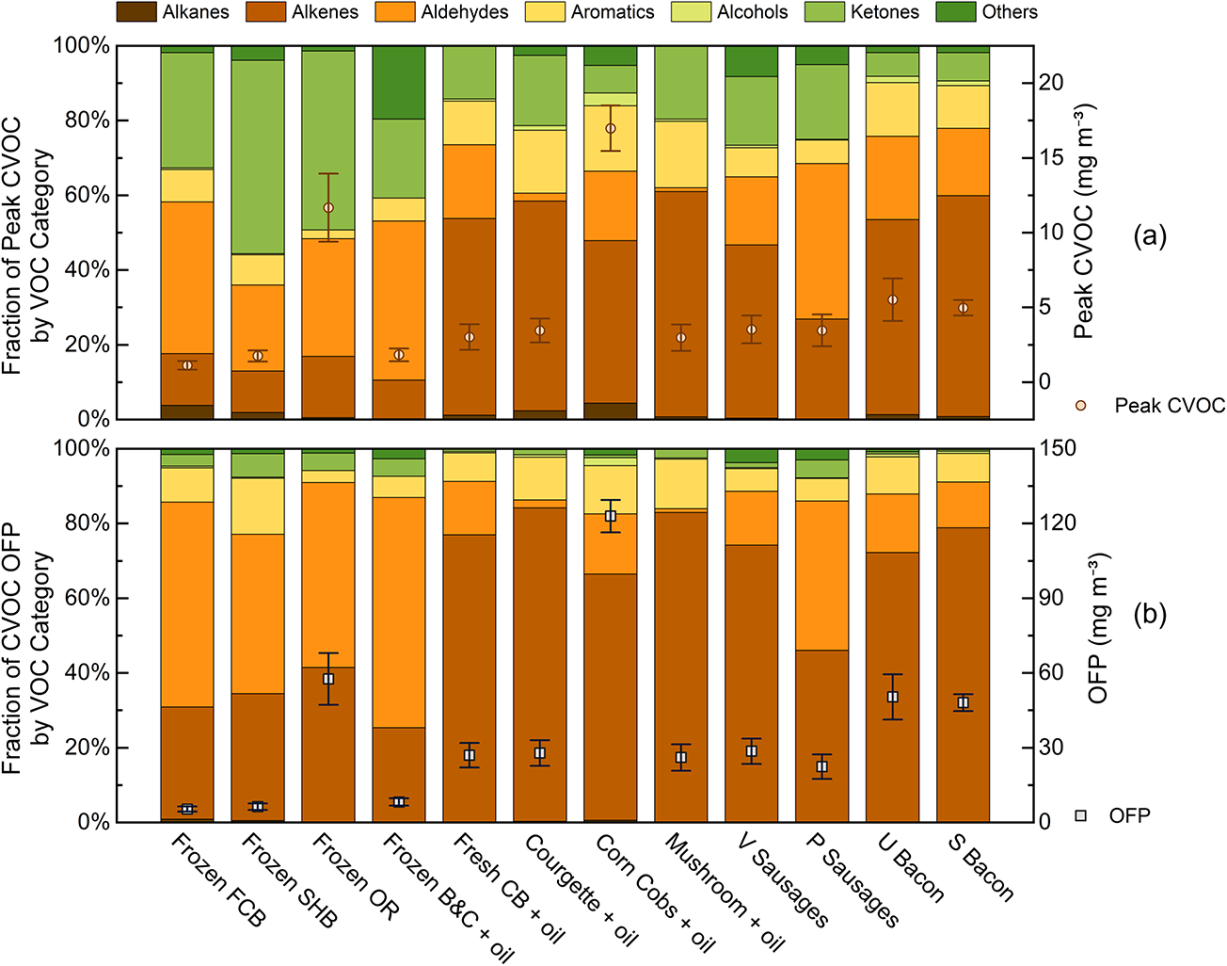
Stacked bar charts of chemical category fractions combined with the dotted graph with standard deviation error bars of (a) peak total CVOC concentrations and (b) OFP concentration for each dish.

Chemically, the dominant VOCs span several classes, as illustrated by the distinct emission profiles shown in the stacked bar charts in Figure 2a. This figure displays the peak VOC concentrations for each dish across various chemical categories including alkanes, alkenes, aldehydes, aromatics, alcohols, ketones, and others. These categories were defined by identifying 22–24 dominant VOCs, which together contribute over 95% of the total CVOCs from each dish. Despite large differences in the total emitted mass, the fractional composition of CVOCs is broadly similar across many dishes. All air-fried foods release a mixture of hydrocarbons, oxygenated compounds and furanic compounds typically associated with heated cooking oils and browned foods due to Maillard reactions.^8,51–53^ In general, ketones (21.1–51.8%) and aldehydes (23.1–42.6%) dominate in terms of total CVOCs from the frozen fried foods and the oiled frozen broccoli and cauliflower, whereas emissions from oiled low-fat and high-fat foods are dominated by alkenes (43.5–60.3%, except for pork sausages at 26.9%).

Specifically, alkanes and small alkenes (C_4_–C_8_) were ubiquitous, as expected for lipids undergoing thermal cracking.^8,17^ For instance, large amounts of 2-butenes (cis and trans isomers) were detected in nearly every dish. These small olefins are well-known products of hot oil vapors and were also prominent in grill/barbecue and frying cooking fumes (i.e., alkenes make up approximately 36% of barbecue CVOC emissions, with 1-butene ranking among the top species).^48^ Meanwhile, oxygenated VOCs (OVOCs)—particularly aldehydes and ketones—are consistently emitted from heated oils and foods. These include typical oil oxidation products such as hexanal and butanone.^8,54^ Across dishes, CVOC ER showed a positive association with relative weight loss (Spearman ρ ≈ 0.6, *p* < 0.05). This compositional profile aligns with known cooking processes: high-temperature heating of oils and fats tends to produce alkenes and carbonyls via the thermal decomposition of fatty acids, whereas Maillard reactions and carbohydrate pyrolysis generate furanic compounds and small aldehydes.^42,53,55,56^ Indeed, the prevalence of 3-methylfuran in emissions from starchy or sugary foods such as corn suggests significant Maillard chemistry as the food surface browns and heated oil is deposited.^56,57^ Furthermore, fatty foods (e.g., bacon and sausages) and oil-rich items (i.e., oiled low-fat foods) released higher amounts of alkenes such as trans-2-butene and 1,3-butadiene, which are likely cracking products of unsaturated lipids.^58–60^ Individual average peak concentrations and OFPs for the dominant VOCs from each dish are provided in the Supporting Information Table S2.

Although most dishes emitted similar types of VOCs and the overall ranking of OFP values mirrors the total CVOC concentrations as well, the relative contributions of individual VOC categories to the OFP varied because of differences in both their quantities and reactivities. The fractions of each VOC category contributing to total OFP from each dish are presented in Figure 2b. In general, alkenes and aldehydes dominated the OFP fractions, while other VOC groups contributed only marginally across all the dishes. This is primarily due to their Maximum Incremental Reactivity (MIR) values, which reflect the ability of each VOC to form ozone.^47,61^ For instance, in frozen fried foods and oiled frozen broccoli and cauliflower, their weighted average MIR values ranged from 3.6 to 4.9 g O_3_/g VOC, whereas the other dishes exhibit higher weighted MIR values, ranging from 6.5 to 9.7 g O_3_/g VOC (Kruskal–Wallis, *p* < 0.05), indicating more reactive compositions of the CVOCs. This aldehyde/alkene-dominant speciation is consistent with chamber/oxidation-flow-reactor (OFR) studies of heated animal fats, which report aldehydes as major nonmethane organic gases and important precursors to oxidized organic aerosol.^62,63^

The average MIR values for the chemical categories, in descending order (mean ± standard deviation, g of O_3_/g of VOC), are as follows: alkenes (12.2 ± 1.0), aldehydes (7.1 ± 0.9), aromatics (6.1 ± 0.8), alcohols (4.6 ± 0.1), others (2.6 ± 1.6), alkanes (1.1 ± 0.3), and ketones (0.8 ± 0.3). This explains why, even though ketones represent a significant fraction of the VOCs in frozen fried foods, their overall contribution to OFP is very weak. In contrast, even if alkenes account for less than 20% of the total CVOCs of the frozen fried foods, they can contribute up to 41% of the total OFP because of their high MIR values. In particular, the high concentrations of trans-2-butene, with an MIR value of 15.16 g O_3_/g VOC,^29^ in oiled low-fat and high-fat foods (except for pork sausages) drives a substantial ozone formation potential. Thus, although pork sausages emit a higher total VOC mass compared to oiled mushrooms, their lower trans-2-butene emissions result in a substantially lower OFP. Conversely, acetone, which is one of the top five emitted VOCs in most of these dishes, contributes little to the OFP due to its low MIR value of only 0.36 g O_3_/g VOC.^19,29,50^ Therefore, while air fryers often use less oil than deep-frying, they can still generate considerable VOC pollution, including reactive VOCs like butenes, furans, aldehydes, and aromatics, which could meaningfully contribute to indoor ozone formation and secondary organic aerosol if not properly diluted.^41,64–66^ In addition, because foods began at different initial temperatures and masses, their effective heating profiles and browning differed despite the fixed 175 °C set point. This helps explain why frozen prepared foods, which largely underwent reheating, showed lower MIR with alkane/ketone-dominated VOCs, whereas hotter cooks produced more alkenes/aldehydes, consistent with prior temperature-dependent cooking chemistry.^67–70^

#### 3.1.2 Emission Dynamics and Source Contribution

To better understand the temporal behavior of VOC emissions and the relative contributions from oil and food, we examined time-resolved concentrations of trans-2-butene (T2B) and 3-methylfuran (3MF). Although our initial Principal Component Analysis (PCA) sought to differentiate ‘oil-driven’ from ‘food-driven’ emissions, the results indicated that both sources largely coemit VOCs. Consequently, we selected T2B and 3MF as representative markers. T2B, a small alkene, is predominantly generated via the thermal cracking or oxidation of unsaturated lipids, whereas 3MF—a heterocyclic compound—arises from both oil degradation and the thermal decomposition of organic food components.^56,59,60,71^ Notably, 3MF formation follows slower kinetics, likely because sustained heating is required to trigger Maillard reactions and protein breakdown.^56,57,71^

Figure 3a–f displays the time series of the averaged concentrations of T2B (blue) and 3MF (red) for five representative dishes alongside an oil-only heating test. The plots span from the moment the air fryer is turned on until 2 min after the concentration peak, with the left dashed line indicating the automatic shut-off and the right dotted line marking the peak. In most dishes, T2B concentrations exceed those of 3MF, confirming its robustness as an indicator of oil-derived emissions. However, in examples such as frozen smiley hash browns and pork sausages, T2B levels are relatively low despite high levels of other oil-derived VOCs (e.g., propene, 1,3-butadiene, and isoprene).^24^ This discrepancy suggests that T2B sensitivity is closely linked to the lipid content: high-fat and oiled foods yield higher T2B emissions, whereas products dominated by Maillard-type reactions tend to exhibit a diminished T2B emission.^55–57,72^

**Figure 3:**
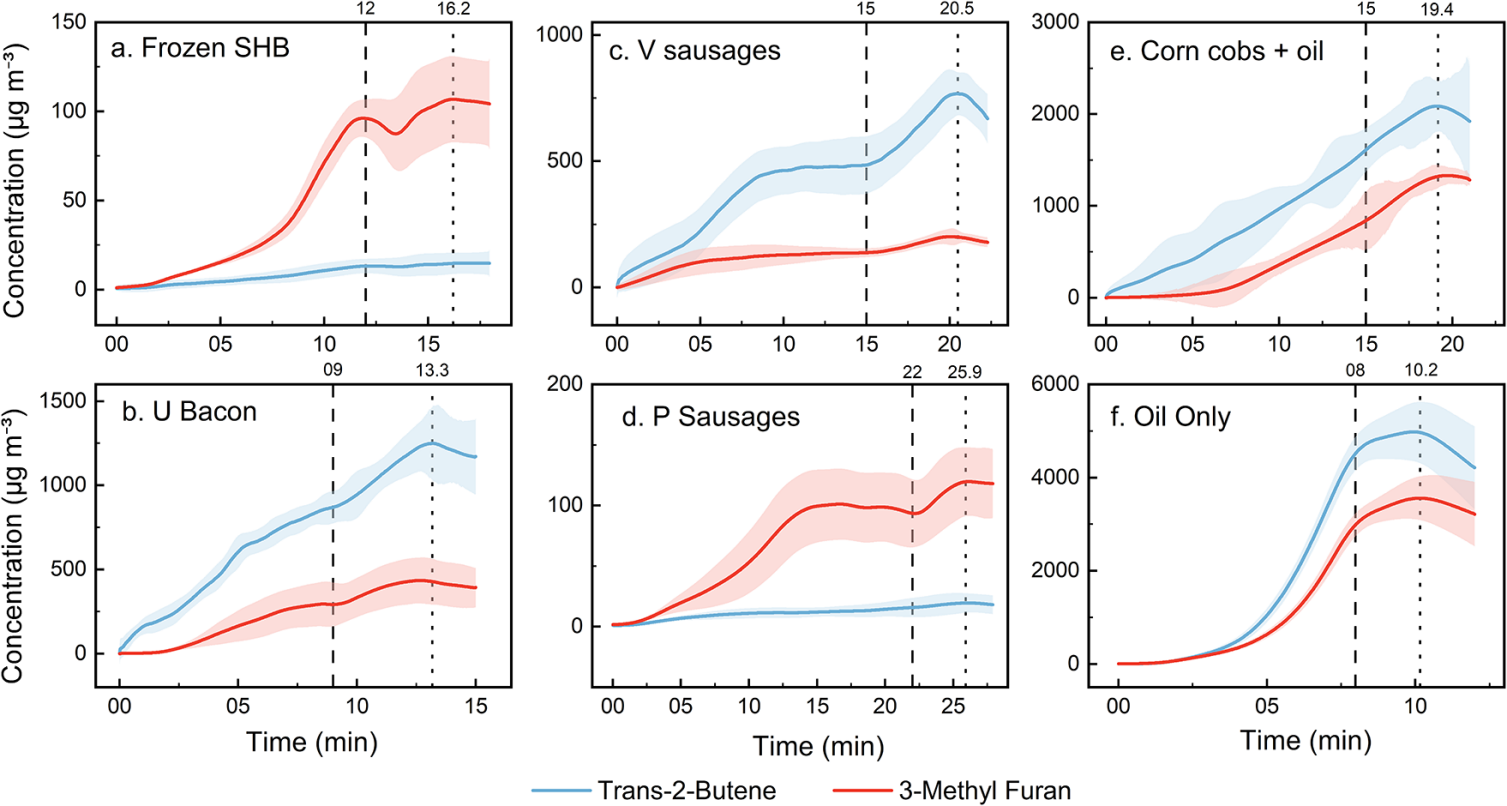
(a–f) Time-series profiles of the mean concentrations (μg m^–3^) of trans-2-butene (blue) and 3-methylfuran (red) emitted from selected dishes. The left dashed line denotes the air fryer off time, and the right dotted line shows the peak concentration time, respectively, in each plot.

The oil-only sample (Figure 3f) illustrates that both T2B and 3MF can be generated solely from heated oil, with their nearly perfect correlation (*r* = 0.998) yielded by Pearson correlation analysis confirming a single source. However, in frozen fried foods, oiled low-fat foods, and high-fat foods, the correlations (*r*) are somewhat lower, ranging 96.2–97.3, 87.6–94.9, and 95.3–98.3%, respectively, suggesting contributions from other constituents and reaction pathways. Notably, the weaker correlations observed in the oiled low-fat foods indicate a stronger contribution from food-derived emissions rather than oil degradation alone. The significantly higher concentrations observed in the oil-only test likely reflect a faster, sustained temperature rise and lower tray humidity, which accelerate oil decomposition. In the presence of food, however, the mass and water content moderate the oil temperature, thereby limiting further thermal breakdown.^70,73^

For low-fat foods with oil sprays applied, T2B exhibits a rapid increase immediately after the air fryer is switched on, while 3MF develops after a 3–5 min delay and increases more gradually thereafter. This pattern suggests that the oil applied to the food surface heats and decomposes swiftly, whereas food-driven reactions require additional time for initiation as surface browning begins. Similar trends are observed in frozen fried and high-fat dishes, where a high oil or fat content produces prompt oil-derived emissions, followed by a delayed onset of food-related reactions as sufficient reactive browning occurs.^8,43,56,57^ In addition, frozen foods exhibit a two-phase emission profile during operation of the air fryer: an initial low-VOC phase dominated by water vapor as the food thaws, followed by a spike in VOC emissions once the food surface dries and the browning reaction accelerates, whereas foods cooked from chilled temperatures tend to begin emitting VOCs immediately once the air fryer is switched on.^52,74^

The emission profiles further highlight the interplay between the air fryer’s operational cycle and the underlying chemical processes. Rapid heating drives early emissions, governed by the dish’s thermal response and the kinetics of reactions such as lipid oxidation and Maillard processes. In several cases, the emission profile slows or plateaus shortly before the air fryer automatically switches off, potentially due to the depletion of highly volatile compounds or the stabilization of the temperature relative to the chamber’s air exchange rate. Moreover, intense heat and prolonged exposure can disrupt cell membranes and denature proteins in fresh ingredients, releasing pro-oxidant species (i.e., reactive intermediates that enhance oxidative reactions) that further promote thermal reactions.^75,76^ Once the air fryer automatically switches off and the internal fan ceases operation, the air exchange rate is reduced to natural diffusion, causing either brief drops or more gradual changes in emissions. These emissions then continue to increase until peaking after 3–6 min, a continued rise that is attributed to the residual high temperature within the air fryer sustaining a thermal response.

#### 3.1.3 Emission Rates (ER) and Emission Factors (EF)

Table 2 presents the emission rates (ER) and emission factors (EF) of CVOCs. As the ER calculations account for different air exchange rates during the “air fryer on” and “air fryer off” phases, Figure 4 illustrates the ERs for each phase, while Table 2 provides the weighted average ERs based on the duration of each phase. The selected VOCs include 1,3-butadiene, isoprene, 3-methylfuran, and trans-2-butene, chosen for their representative characteristics regarding health impacts, high reactivity in terms of OFP, and their role as indicators of the emission source.^5,47,55,60,61,72,77,78^ In particular, isoprene indicates intense thermal decomposition of organic matter, 1,3-butadiene which is a toxic diene marking intensive oil cooking, and trans-2-butene (T2B) and 3-methylfuran (3MF), as discussed in the previous section, serve as the markers of lipid thermal cracking and Maillard browning of sugar with partial oil decomposition, respectively.

**Table 2:** Emission Rates (ER) and Emission Factors (EF) of VOCs Are Shown together with the Relative Weight Loss for Each Dish^a^

			ER (μg min^–1^)					
dish	relative loss in weight	*t*_peak_ (min)	CVOC	1,3-butadiene	isoprene	3-methylfuran	trans-2-butene	EF (μg g^–1^) CVOC
frozen FCB	11.40%	22	17.75	0.78	0.89	0.73	N/A	2.3
frozen SHB	10.00%	16.2	17.56	0.89	0.63	1.06	0.23	1.93
frozen OR	29.50%	18.7	183.98	1.03	0.89	1.19	31.49	22.83
frozen B&C + oil	28.40%	17	24.01	0.58	0.85	0.31	N/A	1.35
fresh CB + oil	29.90%	17.3	36.33	1.6	1.67	2.32	8.95	4.15
courgette + oil	41.50%	14.7	48.96	3.33	3.76	5.13	13.26	4.76
corn cobs + oil	11.80%	19.4	181.4	10.59	12.32	16.23	17.85	9.29
mushroom + oil	16.50%	13.3	42.7	4.58	2.5	0.34	0.74	2.57
V sausages	14.40%	20.5	38.16	1.54	1.79	2.1	9.1	4.29
P sausages	18.50%	25.9	54.52	1.83	5.36	1.5	0.22	6.19
U bacon	46.10%	13.3	108.86	5.2	6.38	6.32	11.38	23.73
S bacon	50.70%	13.9	104.3	3.13	3.91	3.83	14	23.77

a N/A: not applicable due to insignificant emissions of this VOC, while it is present in the ambient air background.

**Figure 4:**
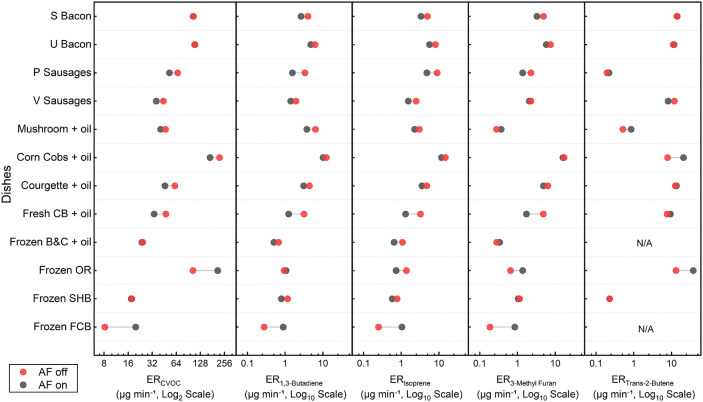
Emission rates (ER, μg min^–1^) of VOCs for each of the dishes when the air fryer is on (black circles) and off (red circles) after cooking (points are paired for each dish).

Emission rates for air frying the various dishes generally reflect their total CVOC concentrations. Frozen fried foods, apart from the onion rings, yield the lowest emission rates (approximately 17 μg min^–1^) due to the much lower temperatures of the ingredients, which take much longer to heat up than the other foods. Although the VOCs begin to form in a pattern similar to that observed for the other dishes, the air fryer switches off before significant emissions are produced, effectively resulting in a reheating process from precooked rather than cooking from raw. Frozen onion rings stand out with exceptionally high emission rates (183.98 μg min^–1^) and rank second in total CVOC concentrations (11.68 mg m^–3^), exhibiting one of the highest EFs (22.83 μg/g), which can be attributed to several factors: (i) the porous batter, enriched with carbohydrates and proteins from the flour and seasonings, allows for efficient transport of volatile compounds, and (ii) the prefried oil coating promotes intense Maillard browning and subsequent oil reheating.^7,52,79^ This is evident in the exceptionally high emission rates of trans-2-butene, a well-known marker of oil/fat decomposition and the thermal breakdown of carbohydrates and lipids.^8,9^ After the air fryer switches off, overall CVOC levels and most individual VOC emission rates drop, highlighting the transient nature of these peak emissions. Moreover, the onion itself is a strong VOC emitter, releasing substantial amounts of acetone, dimethyl disulfide, hexanal, propene, isoprene, pentane, 2-methylfuran, 3-methylfuran, hexane, and other compounds.^80–82^

Within the group of oiled low-fat foods, oiled corn on the cob exhibits an emission rate of 181.49 μg min^–1^, comparable to that of frozen onion rings, by emitting the highest CVOC concentration (16.99 mg m^–3^), while the emission rates of the other dishes in this group range only from 24.01 to 49.96 μg min^–1^. Corn cobs contain substantial natural sugars and amino acids which, upon exposure to high heat, undergo caramelization and Maillard reactions, processes further enhanced by the large surface area of the kernels. Notably, the top part of the cob browns more intensely, because the kernels are cut before packaging, exposing their endosperm to air and heated oil.^83,84^ A study demonstrated that high-temperature treatment such as frying induces lipid oxidation and Maillard reactions in corn, leading to the formation of key VOCs (e.g., *n*-hexanal, 1-octene-3-ol, 2,5-dimethyl-pyrazine) characteristic of fried corn.^85^ Our emission profile of the selected VOCs shows that air frying corn cobs with rapeseed oil promotes the formation of reactive unsaturated hydrocarbons and furans via similar thermal degradation pathways, underscoring the crucial impact of the cooking conditions on both flavor development and potential health aspects, such as the highest emission rate of 1,3-butadiene among all dishes.^52,85,86^ However, the emission factor of corn cobs is lower than that of frozen onion rings and bacon due to their greater overall weight, much of which is contributed by the cob even though the absolute emissions are substantial.

Among other oiled foods, the ER of trans-2-butene of oiled large flat mushrooms is significantly lower (only 0.74 μg min^–1^) compared to other dishes in this food group, for which the ERs typically range 8.95–17.85 μg min^–1^. This difference can be attributed to the juice released from the mushroom, which collected in its canopy when the mushroom was inverted.^87^ As the majority of the oil spray was applied to the bowl-like canopy, the retained juice buffered the temperature, resulting in a much lower emission rate of trans-2-butene. Therefore, due to the melting of ice in the frozen broccoli and cauliflower, the liquid in the air fryer tray buffers the oil sprays on the foods, resulting in no detectable emissions of trans-2-butene.

Among high-fat foods, bacon represents an extreme case and ranks highest in EF among all dishes due to its significantly higher emissions and low weight. It not only generates the highest overall VOC emission rate but also maintains an unusually steady CVOC output even after the air fryer is turned off. By contrast, although both types of sausages produce substantial amounts of VOCs from their rendered grease, their emission rates during air frying are lower than those of bacon, and they tend to show a slightly higher emission rate once the air fryer is turned off. The unique behavior of bacon can be attributed to its cured fat content and thin shape, which promote almost instantaneous frying of lipids throughout its entire mass, thereby releasing a burst of VOCs unmatched by other foods.^88,89^ Additionally, bacon exhibits a significant weight loss during cooking, indicating that intense drying processes lead to considerable evaporation of water and gaseous chemicals, along with the release of melted fat onto the air fryer tray.^73,89^ This residual liquid fat, which results in a larger surface area, continues to emit VOCs even when the air fryer is switched off. Furthermore, because bacon rashers are thin and light, its temperature drops more rapidly than that of sausages under the same conditions, resulting in a nearly constant emission rate during and after air fryer operation, whereas the thicker and heavier sausages cool more slowly, leading to an increase in their emission rate after the air fryer is turned off. The high ER of trans-2-butene and isoprene as well as their high contributions to CVOC and the same pattern of unchanged ER in the two air fryer phases illustrate that the melted fat plays an important role in the continued emissions.^7,89,90^ Again, the extra low emission of trans-2-butene from pork sausages (0.74 μg min^–1^) while the ERs from other high-fat foods ranging from 9.10 to 14.00 μg min^–1^ may also be attributed to the physical barrier provided by the skin of the sausage, as there is not much liquid fat found in the tray. Vegetarian sausages also show a high emission rate of trans-2-butene which is more than a quarter of its total CVOC emission rate due to its internal high oil content.

Although fat-rich foods emit significantly higher levels of VOCs compared to other food types, the overall emission rates of total CVOC from air frying range from only 0.017 to 0.184 mg min^–1^. In comparison, Chen, Zhao^42^ found that deep frying beef with vegetables produced a VOC emission rate of 2.3 ± 0.7 mg min^–1^, and another study reported a rate of 10.9 mg min^–1^ for deep-fried wheat flatbread dough (puri).^43^ A cross comparison of VOC and UFP concentrations and emission rates from different cooking methods reported by past papers is presented in SI Table S3, which highlights that air frying substantially reduces VOC emissions relative to the traditional frying methods and exhibits a similar level of emission rates compared to boiling and steaming, which are considered healthy cooking methods.^42,43,50^ In addition, given that the air fryer also provides an alternative to oven cooking, Kim, Kim^91^ heated corn oil for 120 min using both methods and found that the conventional oven generated roughly 2.3 times higher levels of headspace volatile oxidation products compared to the air fryer. This finding indicated a slower lipid oxidation under air frying, which plausibly reflects thinner oil films, smaller local headspace, steam dilution, and short air-residence times that reduce effective oxygen availability at the hot oil surface.^91–94^ Furthermore, a study determined beef patties cooked in an air fryer had significantly lower benzo­[a]­pyrene levels (BaP, approximately 22.7 ng/kg lower) than those cooked in a conventional oven, with oil-free air frying reducing BaP concentrations to below the detection limit.^23^ Collectively, these findings underscore that the emissions of volatile compounds are strongly influenced by the cooking method, with air frying clearly offering potential advantages in reducing VOCs.

## 4 Nitrogen Oxides (NO_*x*_)

NO_*x*_ levels were monitored alongside VOC and particle concentrations, but only the frozen smiley hash browns and both types of bacon exhibited significant emissions. Table 3 presents the peak concentrations of NO and NO_2_ as well as the NO_*x*_ emission rate. The average chamber concentrations of NO_2_ at baseline and during the cooking of other dishes are 19.6 ± 1.6 and 19.4 ± 2.3 μg m^–3^ (*p* > 0.1), respectively, indicating that neither the operation of the air fryer nor the cooking of the other foods generates significant amounts of NO_2_ or NO. Thus, the observed rise in the NO_*x*_ concentration appears to be food-dependent. Even though the peak NO_2_ concentrations reach approximately 110 μg m^–3^ in the chamber, the estimated real-world peak concentration would be only around 1.1 μg m^–3^, assuming that the same mass of NO_2_ is dispersed in a well-mixed 15 m^3^ kitchen, which is well below the UK Government^95^ annual guideline of 40 μg m^–3^. Given known Mo-converter and humidity artifacts, these NO_2_ values should be interpreted with an uncertainty of roughly +10–30% (occasionally higher) relative to true NO_2_.^34,96^

**Table 3:** Peak Concentrations (μg m^–3^, Mean ± Standard Deviation) of NO and NO_2_ and Emission Rates (ER, ×10^–3^ μg min^–1^) and Emission Factors (EF, × 10^–3^ μg g^–1^) of NO_*x*_ (NO_*x*_ = NO + NO_2_)

	concentration (μg m^–3^)			
dish	NO	NO_2_	ER NO_*x*_ (×10^–3^ μg min^–1^)	EF NO_*x*_ (×10^–3^ μg g^–1^)
frozen SHB	49.1 ± 5.4	104.5 ± 10.9	24.6	2.2
U bacon	12.4 ± 3.1	113.2 ± 14.2	35.3	7.5
S bacon	23.4 ± 6.2	107.7 ± 20.4	37.9	8.1

It is unsurprising that bacon emits substantial amounts of NO_*x*_, since its curing process involves the addition of sodium nitrite (NaNO_2_), which inhibits bacterial growth and, through its conversion to nitric oxide, contributes to these emissions.^97^ Furthermore, the similar peak concentrations and emission rates observed for unsmoked and smoked bacon are consistent with Crowe, Elliott^97^ who reported no significant difference in residual nitrite concentrations between smoked and unsmoked bacon samples. In contrast, the significant emission of NO_*x*_ from the frozen smiley hash browns (“frozen SHB”) may be attributable to the nitrogen contained in potatoes. A study by Rogozińska, Pawelzik^98^ determined that, depending on the potato variety, nitrate and nitrite contents range from 175.4 to 250.7 mg/kg and from 1.7 to 4.3 mg/kg, respectively. These levels can be reduced considerably, 16–62% for nitrates and 61–98% for nitrites, following the cooking process. Additionally, other studies have found that over 85% of the nitrate in potatoes can be reduced during frying,^99,100^ suggesting that the partial loss of these compounds during the cooking process may lead to the formation of NO_*x*_. Figure 5a,b illustrates the time series and correlation between CVOC and NO_*x*_, showing a positive covariation during emitting periods (*p* < 0.05), revealing a strong relationship that demonstrates the coemission of both pollutant types into indoor air. This further supports the conclusion that NO_*x*_ emissions during air frying are food-dependent.

**Figure 5:**
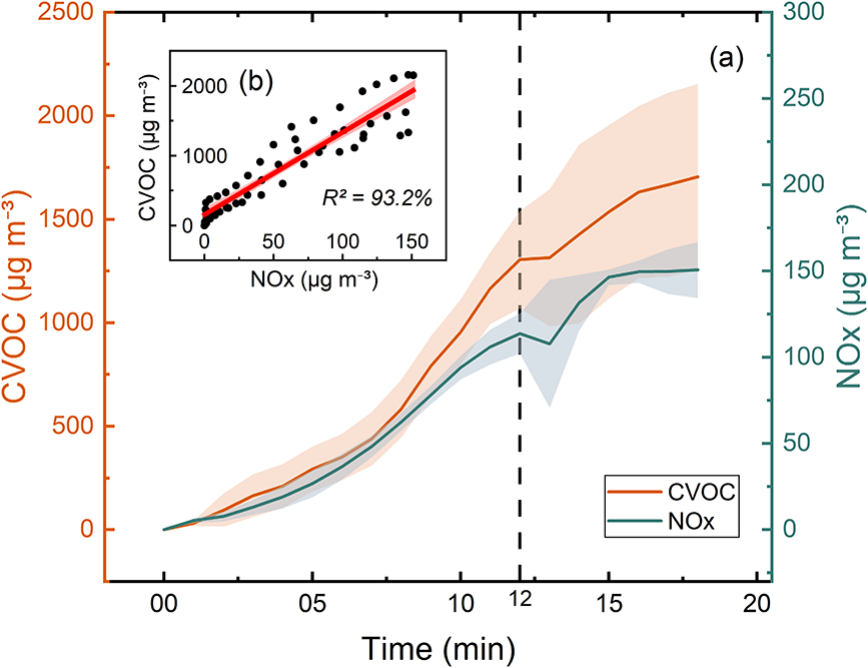
(a) Time series of average concentrations of total CVOC (orange) and NOx (cyan) from air frying frozen smiley hash browns (“frozen SHB”) until their peaks; (b) correlation between these two classes of pollutants from frozen SHB. The air fryer operation for this dish finished at the 12th minute.

## 5 Particles

Air frying the 12 test dishes generates substantial particle emissions with pronounced differences among food categories. Although mass-based PM_1__0/2.__5_ metrics are common, here we report number concentrations of particles ≤300 nm, with UFPs (<100 nm) comprising over 94% of the count. Because aerosol was dried before classification and sampling line losses are minor, no additional correction to the concentration time series was applied. The peak concentrations, emission rates, and emission factors of UFPs from each dish are presented in Table 4. In general, high-fat foods exhibit the highest UFP peak number concentrations (169.5–346.4 × 10^12^ # m^–3^), except the vegetarian sausages, followed by the frozen fried foods (18.6–24.8 × 10^12^ # m^–3^), with exemption of frozen onion rings, while the oiled low-fat foods (2.6–18.1 × 10^12^ # m^–3^) yield the lowest UFP peak number concentrations. While estimating the emissions in a 15 m^3^ kitchen, the peak concentrations are 0.2–34.8 × 10^12^ # m^–3^, consistent with indoor particle dynamics in which ventilation plus deposition substantially lowers room-average UFPs compared with source-proximate or chamber values, as expected from well-mixed mass-balance theory and measured size-resolved deposition indoors.^101,102^ For a representative ducted hood featured larger-kitchen sensitivity (*V* = 30 m^3^, AER = 12 h^–1^, *D*_p_ = 2 h^–1^), the peak concentrations decrease to 0.01–2.49 × 10^12^ # m^–3^, which is more than 10 times lower than the 15 m^3^ case. The strong sensitivity to ventilation + deposition is expected from a well-mixed mass balance. Typical residential AERs are order-unity but can rise by an order of magnitude with a ducted range hood (hundreds of m^3^ h^–1^, substantially reducing room-average UFP.^103–105^ Differences among studies also reflect measurement conditions (room size, hood use, distance, averaging vs peak), which can shift inferred emission rates and peaks by orders of magnitude.^106^

**Table 4:** Peak Number Concentrations (# m^–3^, Mean ± Standard Deviation), Emission Rates (ER, # min^–1^, Mean ± Standard Deviation), and Emission Factors (EF, # g^–1^) of UFP

dishes	concentration (×10^12^)	ER (×10^12^)	EF (×10^9^)
frozen FCB	18.6 ± 3.2	0.9 ± 0.1	98.79
frozen SHB	24.8 ± 1.5	1.3 ± 0.2	103.14
frozen OR	189.0 ± 15.6	9.6 ± 1.2	887.75
frozen B&C + oil	2.6 ± 0.8	0.1 ± 0.03	4.08
fresh CB + oil	8.5 ± 0.5	0.4 ± 0.1	39.79
courgette + oil	18.1 ± 3.6	0.9 ± 0.1	73.36
corn cobs + oil	12.5 ± 0.5	0.6 ± 0.1	24.69
mushroom + oil	8.5 ± 1.6	0.4 ± 0.2	18.29
V sausages	33.2 ± 5.1	1.7 ± 0.4	137.74
P sausages	346.4 ± 10.2	17.4 ± 2.8	1673.66
U bacon	169.5 ± 13.2	9.0 ± 2.7	1318.28
S bacon	255.3 ± 17.6	13.5 ± 3.4	1988.41

This trend of food emission ranking aligns with the known influence of the lipid content on particle formation during cooking, as fatty foods tend to generate more ultrafine particles than lean ingredients.^107,12^ The mechanism involves the vaporization of hot oils and fats, which then rapidly condense to form abundant UFP nuclei, with oil droplet spattering further aerosolizing fine particles.^12,16^ This explains that, except for the frozen onions and the three pork foods, the emission rates are in the range 0.1–1.7 × 10^12^ # min^–1^, which is similar to steaming mutton with vegetable on gas stove at 1.5 × 10^12^ # min^–1^, as reported by Chen, Zhao.^42^ The four high emission foods, by contrast, with emission rates at 9.0–17.4 × 10^12^ # min^–1^ are close to the emission rates of deep frying beef with vegetables and pan frying chicken with vegetables at approximately 9.6 × 10^12^ and 20.2 × 10^12^ # min^–1^,^42^ respectively. Therefore, air frying generates significant UFP emissions that vary markedly with food type: high-fat dishes can produce UFP concentrations comparable to deep frying, while low-fat and oiled items emit considerably lower levels, highlighting the critical influence of the lipid content on particle formation during cooking.

Notable exceptions to the hierarchy above are observed for specific dishes. Despite being grouped as a high-fat dish, the composition of vegetarian sausages, which contain plant-based oils and proteins with a higher moisture content, appears to generate fewer airborne particles. Their emissions match similar particle profiles of those of oiled low-fat foods, which are applied by rapeseed oil sprays. Similar findings are reported that vegetable-based cooking tends to emit lower levels of particles than animal-content based cooking.^108,109^ Furthermore, less oil may be released or combusted from the vegetarian sausages, resulting in a lower nucleation of particles. In the frozen fried foods category, onion rings emit exceptionally high UFP peak concentrations, being even higher than some high-fat foods, such as unsmoked bacon. This outlier behavior is likely because onion rings contain considerable amounts of residual oil in their battered coating from prefrying, and their high surface area facilitates intense aerosolization during air frying. These comparisons underscore that while the drivers of gas-phase (CVOCs) and particulate emissions during cooking are related, they are not identical. Consequently, although both UFP and CVOC emissions depend on the food type, UFP levels are strongly correlated with the amount and the type of oil, whereas CVOC emissions are more influenced by the food temperature and intrinsic properties.

Figure 6A,B illustrates the particle emission dynamics from oiled fresh chicken breast and frozen fried chicken breast, with line graphs highlighting key timings and particle sizes. Both dishes exhibit two to three major emission peaks, which aligns with earlier work that has documented the complex interplay between residual organics on cooking appliance surfaces and the food’s own properties in determining particle formation.^16,107,12,110^ The first peak, emerging around the second minute, is common to both dishes and appears to be linked to residual organic matter on the internal fan and extraction tunnel of the air fryer, which are not accessible for routine cleaning. The second peak, which marks the highest particle concentration, coincides with the cessation of active cooking for the oiled chicken breast. In contrast, the frozen fried chicken breast displays an additional third peak at the very end of the cooking cycle. These differences in peak timing and emission patterns likely reflect the distinct thermal histories of the foods. The fresh chicken, starting from a chilled state, heats steadily, while the frozen chicken must first thaw before cooking, thus prolonging its exposure to heat. During the high-temperature phase, intense fat vaporization and rapid moisture loss promote the condensation of oil vapors into ultrafine particles. A temporary drop in particle concentration for 1–2 min may indicate a momentary depletion of volatile fats or a transient change in airflow that slows nucleation. As cooking continues, further lipid degradation and migration to the surface lead to renewed particle emissions. Finally, when the air fryer is turned off, a final, though lower, peak is observed, likely resulting from the condensation of residual fat vapors during the gradual temperature decline in the absence of active heating.

**Figure 6:**
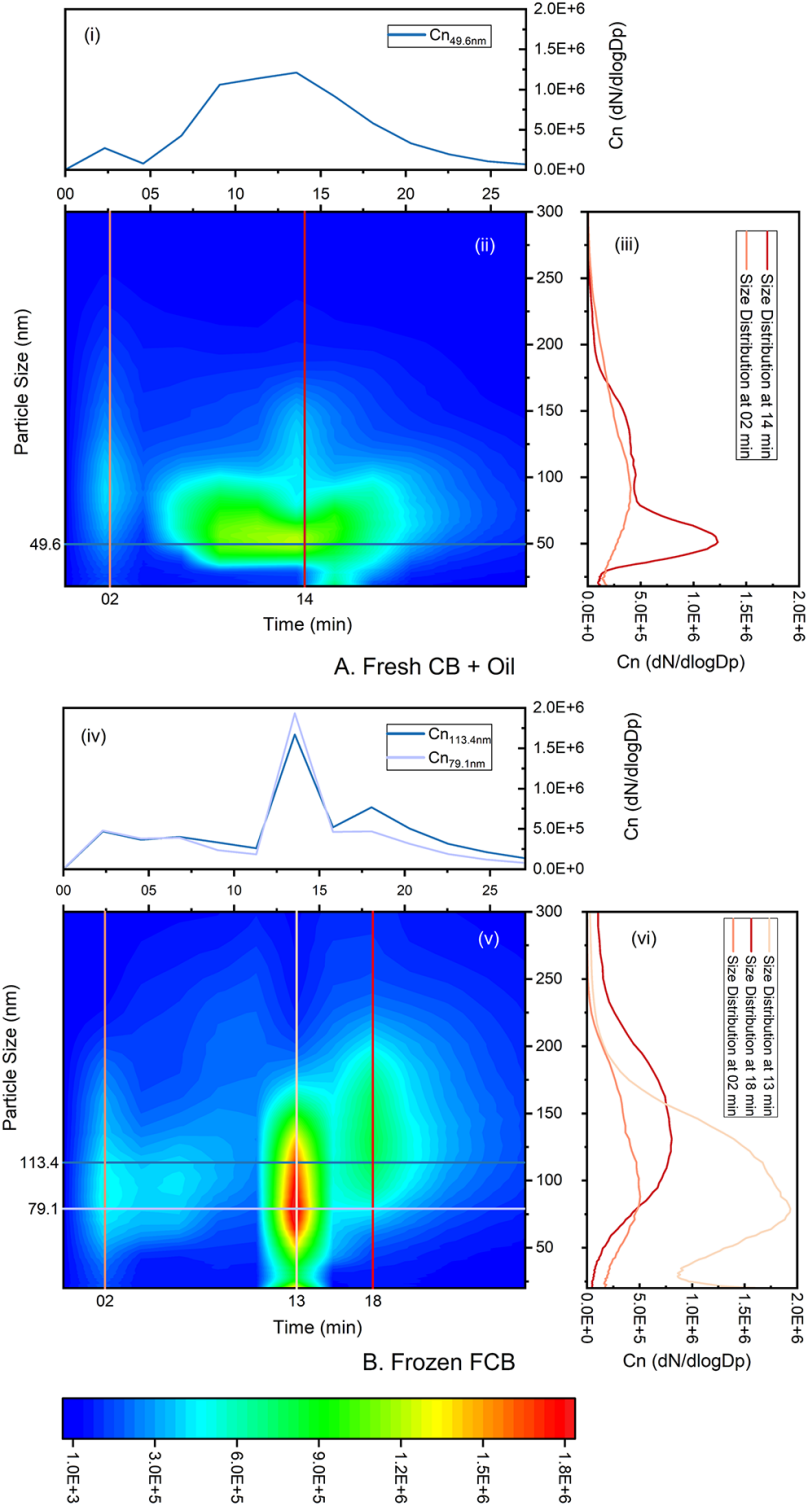
Particle emissions dynamics for (A) oiled fresh chicken breast and (fresh CB + Oil); (B) frozen fried chicken breast (frozen FCB). (A) (i) Time series of particle number concentration (Cn, d*N*/d log *D*_p_) at 49.6 nm (mode of particle size), (ii) contour map of size-resolved number concentration as a function of particle diameter and time, and (iii) particle size distribution at the second minute (first peak) and 14th minute (main peak and end of cooking), as marked by vertical lines in (ii). (B) (iv) Time series of particle number concentration (Cn, d*N*/d log *D*_p_) at 79.1 nm (particle size of the peak) and 113.4 nm (mode of particle size), (v) contour map of size-resolved number concentration as a function of particle diameter and time, (vi) particle size distribution at the second minute (first peak), 13th minute (second peak) and 18th minute (end of cooking), as marked in vertical lines in (v).

While an initial peak due to residual emissions is evident in each dish, it represents another significant source of UFP emissions. Empty air fryer tests were performed at the start of each experimental day. Further repeated empty-run assessments, which were conducted after all dish experiments and an additional campaign (>70 cooking operations in total), revealed that chamber UFP concentrations increased from approximately 7.8 × 10^12^ to 18.4 × 10^12^ # m^–3^, a value exceeding the peak concentrations observed during some cooking runs. Meanwhile, over the same comparison, the total CVOC emissions during empty runs also show a 23% increase. This demonstrates that both particle and gaseous emissions are due to the buildup of residuals in the inaccessible heater/duct areas and not to a within-run time trend. The compounds enhanced in empty-run spectra (propene, acetaldehyde, acetic acid) are typical products of heated oils/fats, and prior work shows that residues on hot kitchen surfaces re-emit when reheated and that empty heated appliances can generate UFP from sorbed organics, not the metal itself. These observations point to oil-film re-entrainment/evaporation in heater/duct regions as the likely reservoir for the +236% UFP increase after heavy use, consistent with nucleation/condensation of semivolatile oil droplets upon reheating.^41,63,69,111,112^ The residues accumulate with use, constitute a noteworthy source of pollution, and raise concerns on the increased personal exposure to pollutants. This finding underscores that residual deposits in hard-to-reach areas not only compromise the air quality benefits of air frying over time but also increase the exposure of the users to pollutants. Designing future air-fryer models with removable or easily accessible cooking-chamber components (i.e., fan housings, heating element shrouds, extraction tunnels) would allow thorough cleaning of all contaminated surfaces, helping to maintain low emissions of both gaseous and particulate throughout the appliance’s lifetime.

In conclusion, air frying emits clearly measurable VOCs, NO_*x*_, and particles—significant in a 0.1518 m^3^ chamber yet, in a real-world kitchen, diluted to levels well below those of traditional frying.^9,42,43,48,50^ Emissions depend on food composition: high-fat and oil-sprayed items produce the most VOCs and particles, while lean meats and frozen foods yield comparatively modest levels. NO_*x*_ arises almost entirely from food rather than from the appliance. Compared with deep- or pan-frying, air frying shows lower emission rates and a weaker ozone formation potential, resembling steaming or boiling. However, residue buildup in the inaccessible areas of the air fryer can elevate emissions over time, so regular cleaning, ideally via fully removable components, is crucial to preserve low-emission performance. With appropriate ventilation and maintenance, air frying stands out as a viable, lower-emission cooking method that can improve indoor air quality and reduce environmental impact in domestic settings. Designs that facilitate access to heater/duct paths would further limit residue-driven increases and support low emissions in routine use. These results emphasize the potential of air frying as a lower-emission alternative that could contribute to improved indoor air quality, provided that users maintain proper cleaning protocols to mitigate secondary emissions from accumulated residues. While the WHO sets no numeric guideline values for total VOCs or UFPs (the EU’s 2024 ambient directive introduces UFP monitoring, but no limits), our kitchen-scale NO_2_ peaks (∼1.1 μg m^–3^) are well below the WHO 24 h guideline (25 μg m^–3^) and our estimated total VOC levels remain below the UK 8-h guidance of 0.3 mg m^–3^, indicating low benchmark exceedance under the scenarios considered.^49,113,114^ Our study suggests that with regular maintenance and appropriate ventilation, the reduction in cooking emissions afforded by air frying can make it a viable and more environmentally friendly option compared with traditional frying methods, particularly in domestic settings where indoor air quality is of increasing concern.

The color scale indicates the particle number concentration (d*N*/d log *D*_p_) in all contour plots.

## Supplementary Material



## Data Availability

The data supporting the conclusions of the study will be made available by the corresponding author upon request.

## Notes

Dimensions and further information on chamber and air fryer; protocols for the cooking experiments and cleaning; the particle rescaling from the chamber to a 15 m^3^ kitchen; the PCA workflow, selection criteria, and quantification; the nutrient composition of the tested foods; individual average peak concentration and OFP values for the dominant VOCs from each dish; and the comparison of cooking-induced VOC concentrations, emission rates of cooking-VOC and UFP, and OFP (PDF)
