# Supplementary material for: Quantification of Volatile Organic Compounds (VOCs), Nitrogen Oxides (NO x ), and Ultrafine Particles (UFPs) Emitted by Domestic Air Fryers: A Chamber Study of Indoor Air Quality Impacts
Source: ACS EST Air. 2026 Jan 27;3(2):473–87. doi: 10.1021/acsestair.5c00363 (PMC12910553; doi:10.1021/acsestair.5c00363)
Supplement: Supplementary file 1 [file ea5c00363_si_001.pdf]

Supplementary Information

Quantification of Volatile Organic Compounds (VOCs), Nitrogen Oxides (NO_x) and Ultrafine Particles (UFPs) Emitted by Domestic Air Fryers: a Chamber Study of Indoor Air Quality Impacts

Ruijie Tang[1], Yizhou Su[1], William Joe F. Acton[1], Lara K. Dunn[1] and Christian Pfrang[1,2,*]

[1] School of Geography, Earth and Environmental Sciences, University of Birmingham, Edgbaston, B15 2TT Birmingham, UK

[2] Department of Meteorology, University of Reading, Whiteknights, Earley Gate, RG6 6BB Reading, UK

*Corresponding Author: c.pfrang@bham.ac.uk

Text S1. Dimensions and further information on the chamber and air fryer.

All cooking experiments were conducted inside a custom-built Perspex chamber (45 × 45 × 75 cm, approx. 151.9 L, supplied by The Plastic People, Leeds, West Yorkshire, UK) for capturing and measuring emissions in a controlled environment. The chamber was constructed to include a 12 V DC brushless fan (RS Components, Corby, Northants, UK), airtight seals, and a removable lid, ensuring a well-mixed internal atmosphere with minimal leakage. The lid was positioned with a 1 cm gap to allow the instrumentation sampling tubing to be fed into the chamber and located in the central area with a distance of approximately 25 cm from the lid and to maintain balanced internal pressure due to the operation of the air fryer, which elevated the temperature and increased the relative humidity. The chamber was placed in a ventilated laboratory in the Biosciences building at the University of Birmingham, UK.

A COSORI air fryer (model CAF-L501-KUK, USA), with 4.7 L capacity and dimensions of 27.2 × 27.5 × 30.3 cm, was placed at the centre of the chamber for all tests. The air fryer has a top-mounted heating element and an internal fan, which circulates hot air to cook the food. Its air outlet is located at the rear, at a height of approximately 18 cm, where the heated air exhausts by diffusion. To optimise air mixing within the chamber, the chamber's fan was positioned at the same height as the air fryer's outlet.

Text S2. Protocols of cooking experiments and cleaning.

S2.1 Cooking experiments

A selection of 12 different dishes commonly consumed in the UK was obtained from local supermarkets to provide a representative range of foods suitable for air frying. These included items across various categories, such as frozen fried foods, fresh and cured meats, and vegetables, which are also commonly chosen for frying or oven-cooking, and represent both high-fat and low-fat options. For items with very low initial fat content (e.g. vegetables and lean meats), a standardised application of five sprays of rapeseed oil (approx. 0.86 g) was applied to the food surface before cooking. This small amount of oil was sufficient to ensure proper browning and realistic cooking conditions for low-fat foods, while maintaining consistency in added fat across the relevant dishes. The weight of each food was recorded before and after cooking, and the cooking time for each dish was initially set according to the respective package instructions or verified online recipes. However, to account for differences among air fryer models and to maintain a uniform cooking temperature across the experiments, the temperature was fixed at 175 °C in our study. Consequently, cooking durations were adjusted from the instructions based on preliminary tests.

At the start of each experimental day, the air fryer was preheated (empty) at 205 °C for 4 minutes, following the manufacturer's manual, as preheating served to warm the fryer to steady operating conditions and ideally burn off residual volatiles from previous use. After preheating, an air fryer emission test was conducted at 175 °C for 9 minutes with an empty tray to quantify emissions from the air fryer itself. These blank runs established the baseline levels of VOCs, NOx, and particles attributable to the heated appliance and residual deposits that could not be completely cleaned. Additionally, emissions from air frying only five oil sprays were measured at 175 °C for 9 minutes as well.

For each cooking trial, a single dish was placed in the tray-like fry basket and inserted into the preheated air fryer. Due to the necessity for manual operation, the chamber lid was immediately closed as soon as the air fryer was turned on. The air fryer's built-in timer and thermostat controlled the heating process; therefore, no manual intervention occurred during cooking. When the set cooking cycle finished, the air fryer turned off automatically, and the chamber lid remained closed for an additional 10 minutes before any opening. This allowed the immediate post-cooking emissions to mix thoroughly within the chamber until peak and started to decay, which could be fully captured in the measurements. After removing the lid, the air fryer was removed from the chamber, and the cooked food was taken out of the fry basket and weighed again. The chamber and air fryer were then left open for 15 minutes to allow ventilation and cleaning where approachable before starting the next experiment. No preheating was performed between cooking experiments on the same day, as the retained heat from the previous run was sufficient to bring the appliance quickly back to the set temperature.

S2.2 Cleaning protocols of the air fryer and the chamber

Air fryer. Immediately after each run, the tray and rack in the tray were removed while warm (using heat-safe gloves), hand-washed thoroughly with standard dishwashing liquid and warm water, rinsed, and dried with a lab blue towel. The internal walls and cavity were wiped with a damp laboratory cleaning pad; a lab blue towel was then used to dry as needed. In practice, active drying was often unnecessary because the cavity and heating compartment remained hot and self-dried rapidly. Exterior surfaces were wiped to remove visible residues, and the heating area was visually checked for loose debris.

Chamber (after each run). The chamber interior (walls, floor, and lid underside) was wiped with a damp laboratory cleaning pad and then dried with a lab blue towel. The lid was removed and the chamber was left open to the laboratory room to ventilate and cool prior to the next experiment.

Baseline verification before the next experiment. Before starting a new run, we verified that background levels had been re-established. A short SMPS check confirmed particle number concentrations had returned to room background. PTR-MS and NOx analyzers operated continuously, allowing direct confirmation from instrument displays that gas-phase signals had returned to baseline. New experiments proceeded only once all instruments indicated background conditions.

Notes. For safety and design reasons, the heating compartment and internal ducting of the air fryer are not fully accessible for manual cleaning. As discussed in the manuscript, residues can accumulate in these areas over repeated use; this limitation likely explains the "empty-run after heavy use" signal increases and motivates the maintenance/usage implications noted in the discussion. We avoided scented cleaners or sprays, used heat-safe handling at all times, and observed no residual carry-over above background with the protocol described here.

Text S3. Particle rescaling from chamber to a 15 m³ kitchen.

Objective. Provide a compact, transparent rescaling of chamber-derived particle emission rates (ER) to an illustrative 15 m³ kitchen, while acknowledging room-specific uncertainty. We present a median case and a small parameter sensitivity.

Model. We use a well-mixed first-order box model with ventilation and deposition losses:

$$\frac{dC_{in,p}(t)}{dt} = \frac{S_p}{V} - (a + D_p)C_{in,p}(t)$$

where C is the number concentration), $S_p$is the particle number emission rate (# min^{-1} or # s^{-1}), $V$is room volume (here 15 m³), a is the air change rate (h^{-1}), and D_p is the size-resolved deposition loss rate (h^{-1}). Results are shown for total number using the chamber-measured emission rates; size-resolved use follows identically.

Peak (transient) approximation. For short cooking events where ER ramps and then falls, a practical upper-bound for the kitchen peak is

$$C_{\mathrm{peak}} = \frac{ER_{\mathrm{max}}}{V(\mathrm{a} + D_p)}$$

Text S4. PCA workflow, selection criteria, and quantification.

We applied PCA (Varimax rotation) to 326 PTR-MS m/z time series (1 s data averaged to 15 s to reduce noise). For each principal component (PC), we inspected the three highest-loading ions against the time series to determine whether the PC tracked cooking activity (preheat/active cook/post-cook) or reflected background/noise. PCs without clear cooking signatures were discarded. For retained PCs, all ions with loading > 0.6 were compiled as cooking-related VOCs (CVOCs) for downstream analysis (species assignment, time series, peak time/level, ppb→μg m^{-3} conversion, ER/EF, and OFP).

Quantification & identification. Where compound standards/response factors were available, we used compound-specific calibrations; otherwise we applied default/class sensitivities (reflected in the stated PTR-MS uncertainty). ppb → μ g m^{-3} conversion used observed chamber P/T (Section 2.3.1). Potential fragmentation (notably for aldehydes/alkenes) was considered when assigning identities.

Table S1. Nutrient composition of test foods. Values are reported per 100 g (left block) and scaled to the cooked mass used in each experiment (right block).

Dishes	Abbreviation	Every 100 g			Scale to cooked mass		
		Fat	Protein	Carbohydrate	Fat	Protein	Carbohydrate
Frozen Fried Chicken Breast	Frozen FCB	14.0	14.0	21.0	23.8	23.8	35.7
Frozen Smiley Hash Brown	Frozen SHB	9.1	2.5	28.0	13.4	3.7	41.2
Frozen Onion Rings	Frozen OR	10.0	4.1	30.4	15.1	6.2	45.8
Frozen Broccoli and Cauliflower with Oil Sprays*	Frozen B&C +oil*	0.2	2.3	2.0	0.6	6.9	6.0
Fresh Chicken Breast Slices with Oil Sprays*	Fresh CB +oil*	3.3	21.5	0.0	5.0	32.5	0.0
Courgette Slices with Oil Sprays*	Courgette +oil*	0.4	1.8	1.8	0.6	2.7	2.7
Corn on the Cobs with Oil Sprays*	Corn Cobs +oil*	2.5	3.7	16.1	9.6	14.2	61.7
Mushrooms with Oil Sprays*	Mushroom +oil*	0.2	1.0	0.3	0.4	2.2	0.7
Vegetarian Sausages	V Sausages	9.9	14.0	15.0	18.0	25.5	27.3
Pork Sausages	P Sausages	25.1	12.6	11.4	57.3	28.8	26.0
Unsmoked Bacon	U Bacon	13.2	17.4	0.5	8.1	10.6	0.3
Smoked Bacon	S Bacon	13.2	17.4	0.5	8.1	10.6	0.3
Rapeseed Oil Spray (100ml)	Oil	49.5	N/A	N/A	0.5	N/A	N/A

* For oiled low-fat dishes, the reported nutrients are for the food itself excluding the added oil sprays.

Table S2. Individual average peak concentration (Peak C, µg m^{-3}) and OFP (µg m^{-3}) values for the dominant VOCs from each dish ranking by concentrations from high to low.

(a) Frozen fried chicken breast

Chemicals	Peak C	OFP
acetaldehyde	413.88	2706.75
Acetone	295.65	106.43
1,3-butadiene	52.59	663.21
Isoprene	52.35	555.42
3-Methyl Furan	40.48	279.28
methyl ethyl ketone	22.91	33.91
1,2-Dimethyl Cyclohexene	20.58	115.87
phenol	20.16	55.64
terpene (monoterpenes)	19.56	79.02
2-ethyl furan	15.94	113.04
C_{11} Bicycloalkanes	14.51	13.21
C9 bicycloalkane(s)	14.40	20.02
formaldehyde	12.36	116.90
Fragment of Allene (C_3H_4)	11.86	138.28
C5 alkenes	10.64	94.08
3-Methylbutanal	8.02	39.84
C_7 Alkyl Phenols	6.90	16.56
C_{10} Bicycloalkanes	6.53	7.12
C9 ketones	6.05	6.53
Cyclohexane	4.94	6.17
C9 monosubstituted benzenes	4.83	9.81
α-terpineol	4.71	21.81
Dihydroxyacetone	4.62	18.44
C10 alkyl phenols	4.45	7.69

(b) Frozen smiley hash browns

Chemicals	Peak C	OFP
Acetone	825.07	297.03
acetaldehyde	341.25	2231.78
3-Methyl Furan	109.44	805.12
1,3-butadiene	83.67	1055.09
Isoprene	57.93	614.64
dimethyl sulfide (DMS)	57.28	34.37
formaldehyde	36.52	345.52
methyl ethyl ketone	29.48	43.63
C_{11} Bicycloalkanes	26.26	23.90
1,2-Dimethyl Cyclohexene	18.31	103.10
trans-2-butene	15.53	235.45
C5 Ketones (2-Pentanone, 3-Pentanone)	14.59	41.00
3-Methylbutanal	11.21	55.71

phenol	11.19	30.87
2-ethyl furan	11.18	79.24
Trimethylamine	7.31	46.21
C5 alkenes	6.73	59.46
C9 bicycloalkane(s)	5.47	7.61
C9 ketones	5.13	5.54
Cyclopentadiene	4.66	32.56
C_7 Alkyl Phenols	4.65	11.15
α-terpineol	4.33	20.03

(c) Frozen onion rings

Chemicals	Peak C	OFP
Acetone	5095.26	1834.29
acetaldehyde	2029.61	13273.67
formaldehyde	1549.17	14655.12
Propene	1167.07	13608.05
trans-2-butene	525.87	7797.89
3-Methyl Furan	155.58	1472.38
hydroxy acetone	136.49	440.88
methyl ethyl ketone	114.35	169.24
Isoprene	101.11	1072.79
Trimethylamine	96.28	608.49
1,3-butadiene	78.53	990.21
Cyclohexanone	64.14	86.59
C_{11} Bicycloalkanes	59.07	53.76
Dimethyl sulfide (DMS)	58.06	34.83
C7 cyclic ketones	38.47	45.39
phenol	34.02	93.91
C10 alkyl phenols	32.93	56.96
C5 Ketones (2-Pentanone, 3-Pentanone)	31.82	89.40
3-Methylbutanal	22.97	114.18
2-ethyl furan	22.67	160.74
n-pentyl benzene	17.45	37.00

(d) Frozen Broccoli and Cauliflower with Oil Sprays

Chemicals	Peak C	OFP
acetaldehyde	729.32	4769.77
dimethyl sulfide (DMS)	344.03	206.42
Acetone	198.67	71.52
methyl ethyl ketone	135.25	200.17
Isoprene	75.76	803.85
phenol	61.05	168.50
1,3-butadiene	52.17	657.90
Propene	44.41	517.84
C5 Ketones (2-Pentanone, 3-Pentanone)	33.31	93.60

3-Methyl Furan	34.28	232.48
formaldehyde	21.08	199.44
1,2-Dimethyl Cyclohexene	9.03	50.83
2-ethyl furan	7.57	53.66
C9 ketones	4.23	4.57
Hexanal	4.23	18.40
dimethyl amine	3.76	11.91
benzene	3.67	2.64
methyl cyclopentane	3.36	7.37
C9 monosubstituted benzenes	2.56	5.20
Cyclopentadiene	2.46	17.18
Hydroxyacetone	2.42	7.82

(e) Fresh Chicken Breast Slices with Oil Sprays

Chemicals	Peak C	OFP
trans-2-butene	918.84	13929.62
acetaldehyde	553.66	3620.93
Acetone	348.81	125.57
3-Methyl Furan	232.70	1629.00
Isoprene	160.31	1700.86
1,3-butadiene	156.33	1971.35
Propene	193.89	2260.79
phenol	61.39	169.45
1,2-Dimethyl Cyclohexene	59.58	335.45
2-ethyl furan	28.23	200.12
C9 ketones	26.71	28.85
C_{11} Bicycloalkanes	23.17	21.08
formaldehyde	21.39	202.33
α-terpineol	17.48	80.93
C5 alkenes	16.00	141.42
C_7 Alkyl Phenols	15.88	38.12
methyl ethyl ketone	15.72	23.26
C7 cyclic ketones	13.99	16.51
Cyclopentadiene	13.76	96.03
C12 alkenes	12.28	29.35
C9 bicycloalkane(s)	10.18	14.16
C8 cyclic ketones	8.57	9.00

(f) Courgette Slices with Oil Sprays

Chemicals	Peak C	OFP
trans-2-butene	1004.96	15235.22
Acetone	469.80	169.13
3-Methyl Furan	336.39	2321.10
Isoprene	284.60	3019.64
1,3-butadiene	241.77	3048.77

1,2-Dimethyl Cyclohexene	159.12	895.83
phenol	128.18	353.78
C_{11} Bicycloalkanes	59.13	53.81
dimethyl sulfide (DMS)	83.60	50.16
2-ethyl furan	54.19	384.17
formaldehyde	48.71	460.76
C9 ketones	47.81	51.64
methyl ethyl ketone	44.26	65.51
α-terpineol	40.74	188.63
C5 alkenes	81.06	496.61
C_7 Alkyl Phenols	29.91	71.78
C12 alkenes	29.44	70.36
C7 cyclic ketones	28.15	33.22
Cyclopentadiene	26.85	187.39
C5 Ketones (2-Pentanone, 3-Pentanone)	22.94	64.46
3-Methylbutanal	20.46	101.70
C9 bicycloalkane(s)	17.80	24.74

(g) Corn on the Cobs with Oil Sprays

Chemicals	Peak C	OFP
acetaldehyde	2939.86	19226.70
trans-2-butene	2276.27	34508.25
3-Methyl Furan	1442.11	10407.83
Propene	1182.70	13790.26
Isoprene	1079.14	11449.63
1,3-butadiene	868.98	10957.86
1,2-Dimethyl Cyclohexene	740.04	4166.45
phenol	715.75	1975.48
C_{11} Bicycloalkanes	565.74	514.83
Acetone	555.55	200.00
α-terpineol	540.25	2501.37
C9 ketones	480.47	518.91
terpene	433.78	1752.49
dimethyl sulfide (DMS)	393.11	235.87
C12 alkenes	392.05	937.01
2-ethyl furan	342.28	2426.78
C_7 Alkyl Phenols	263.79	633.11
C5 alkenes	178.87	1581.25
Cyclopentadiene	172.62	1204.87
C8 cyclic ketones	133.92	140.61
C9 bicycloalkane(s)	125.02	173.77

(h) Mushrooms with Oil Sprays

Chemicals	Peak C	OFP
trans-2-butene	699.07	10597.87

Isoprene	369.12	3916.41
Fragment Propene (C_3H_6)	265.83	3099.61
Acetone	251.05	90.38
3-Methyl Furan	221.19	1691.39
C8 Aromatics (Ethylbenzene, Xylenes)	174.99	1336.90
methyl ethyl ketone	172.19	254.84
1,3-butadiene	167.16	2107.87
C5 Ketones (2-Pentanone, 3-Pentanone)	86.95	244.33
1,2-Dimethyl Cyclohexene	86.61	487.62
phenol	67.40	186.04
Propene	58.16	678.20
formaldehyde	28.47	269.29
C9 ketones	24.66	26.63
2-ethyl furan	24.39	172.90
C_{11} Bicycloalkanes	20.63	18.77
C5 alkenes	39.31	249.56
C7 cyclic ketones	18.54	21.88
α-terpineol	15.58	72.15
Cyclopentadiene	14.15	98.79
benzene	12.46	8.97

(i) Vegetarian Sausages

Chemicals	Peak C	OFP
trans-2-butene	887.39	13452.82
acetaldehyde	557.61	3646.77
Acetone	519.05	186.86
Propene	318.59	3714.72
Isoprene	158.55	1682.25
3-Methyl Furan	167.24	1297.45
1,3-butadiene	133.92	1688.67
terpene	257.68	1041.03
methyl ethyl ketone	55.93	82.78
phenol	51.96	143.41
formaldehyde	37.05	350.53
2-ethyl furan	31.30	221.89
1,2-Dimethyl Cyclohexene	26.62	149.88
C9 ketones	22.27	24.05
C5 Ketones (2-Pentanone, 3-Pentanone)	21.69	60.94
α-terpineol	20.26	93.79
Dimethyl sulfide (DMS)	19.32	11.59
3-Methylbutanal	16.46	81.80
C12 alkenes	13.66	32.65
C_7 Alkyl Phenols	13.60	32.63
C_{11} Bicycloalkanes	13.12	11.94
C5 alkenes	11.55	102.14
Cyclopentadiene	8.69	60.69

(j) Pork Sausages

Chemicals	Peak C	OFP
acetaldehyde	1198.62	7838.99
Isoprene	382.14	4054.51
methyl ethyl ketone	344.96	510.54
Propene	334.54	3900.75
C5 Ketones (2-Pentanone, 3-Pentanone)	176.81	496.83
3-Methylbutanal	166.05	825.26
1,3-butadiene	141.06	1778.71
Acetone	133.40	48.02
terpene	154.72	625.06
3-Methyl Furan	122.65	991.86
phenol	52.70	145.44
2-ethyl furan	24.38	172.85
trans-2-butene	20.31	307.83
Hexanal	12.52	54.46
1,2-Dimethyl Cyclohexene	11.44	64.42
C9 ketones	10.87	11.74
formaldehyde	10.61	100.35
C_7 Alkyl Phenols	9.14	21.94
C5 alkenes	8.15	72.06
α-terpineol	6.77	31.33
2-(2-Butoxyethoxy) Ethyl Acetate	6.57	9.06
dimethyl amine	6.52	20.65

(k) Unsmoked Bacon

Chemicals	Peak C	OFP
trans-2-butene	1361.61	20641.96
acetaldehyde	1164.34	7614.81
Propene	463.89	5409.00
3-Methyl Furan	459.55	3598.35
Isoprene	393.15	4171.31
1,3-butadiene	301.22	3798.42
Acetone	163.67	58.92
phenol	152.26	420.25
2-ethyl furan	100.33	711.32
1,2-Dimethyl Cyclohexene	96.33	542.35
C9 ketones	95.67	103.32
Terpene	93.70	378.53
α-terpineol	88.09	407.85
C12 alkenes	61.22	146.33
C_{11} Bicycloalkanes	46.14	41.98
methyl ethyl ketone	35.20	52.10
C_7 Alkyl Phenols	34.74	83.37

Cyclopentadiene	26.94	188.03
C9 bicycloalkane(s)	23.01	31.98
C5 Ketones (2-Pentanone, 3-Pentanone)	19.94	56.03
C5 alkenes	19.82	175.22
C7 cyclic ketones	18.88	22.28

(l) Smoked Bacon

Chemicals	Peak C	OFP
trans-2-butene	1602.03	24286.72
acetaldehyde	841.22	5501.56
Propene	546.75	6375.09
Isoprene	295.60	3136.35
3-Methyl Furan	342.10	2360.46
1,3-butadiene	231.87	2923.91
Acetone	268.55	96.68
phenol	105.87	292.20
2-ethyl furan	73.09	518.20
C9 ketones	64.72	69.90
1,2-Dimethyl Cyclohexene	61.75	347.65
α-terpineol	59.67	276.27
C12 alkenes	39.38	94.11
methyl ethyl ketone	25.45	37.67
C_7 Alkyl Phenols	23.37	56.08
C_{11} Bicycloalkanes	22.87	20.82
Cyclopentadiene	18.31	127.82
C9 bicycloalkane(s)	17.00	23.64
C5 alkenes	16.84	148.84
terpene (monoterpenes)	87.60	90.72
formaldehyde	14.50	137.13

Table S3. Comparison of cooking-induced VOC concentrations, emission rates of cooking-VOC and UFP, and OFP.

Study	Volume of room or chamber (m^3)	Heating source	Cooking method	Ingredients		Oil		Cooking-VOC Concentrations (mg/m^3)	Average emission rate		OFP (mg/m^3)
				Type	Weight (g)	Type	Amount		Cooking-VOC (mg/min)	UFP ($\times 10^{12}$ #/min)	
Zhang, Wang, Shen, Li, Wu, Li, Bai, Cao, Hao, Zhou and Yao [1]	N/A (Pipeline Sampling)	Electric hob	Braising and frying	Meat with vegetable		Blend		1.8 ± 0.3			4.85–8.29
		Electric hob	Stewing, frying and steaming	Meat with vegetable		Blend		2.3 ± 0.7			
		Charcoal fire	Barbecue	Meat		Blend		14.4 ± 6.9			
		Electric hob	Frying	Meat with vegetable		Blend		2.7 ± 0.5			
Chen, Zhao and Zhao [2]	10.88	Gas stove	Boiling	Vegetable	120	Soybean			0.4 ± 0	1.4 ± 0	
			Deep-frying	Beef with vegetable	120	Blend			2.3 ± 0.7	9.6 ± 1.8	
			Pan-frying	Chicken with vegetable	120	Canola			4.5 ± 0.8	20.2 ± 0.1	
			Steaming	Mutton with vegetable	120	Sunflower			0.2 ± 0.1	1.5 ± 0	
			Stir-frying	Fish with vegetable	120	Peanut			5.1 ± 2.3	23.2 ± 0.4	
Cheng, Wang, Lang, Wen, Wang and Yao [3]	N/A (Pipeline Sampling)	Natural Gas	Stir-frying, stewing and steaming			Salad		0.49	94.14		1.95
		Natural Gas	Mixed			Soyabean		0.26	29.32		1.11
		Liquefied Gas	Mixed			Salad		0.29	79.48		2.01
		Fruit Charcoal	Barbecue			Salad		3.49	85.71		16.82
Kumar, O'Leary, Winkless, Thompson, Davies, Shaw, Andrews, Carslaw and Dillon [4]	15	Electric cooker	Deep-frying	Wheat flatbread dough (puri)	10	Rapeseed	100 mL		10.9		
			Frying	Garlic	10	Rapeseed	10 mL		2.3		
			Frying and stewing	Chicken curry with spices and herbs	582	Rapeseed	10 mL		2.0		
This study	0.1518	Air-fryer	Air-frying	Frozen fried foods	147.3–170	No added oil	N/A	1.1–11.7* (0.01–0.12)**	0.02–0.18	0.9–1.3 Frozen OR: 9.6	5.4–57.6* (0.06–0.58)**
			Air-frying	Oiled low-fat foods	151.3–383.3	Rapeseed	0.86 g	1.8–17.0* (0.02–0.17)**	0.02–0.18	0.1–0.9	8.2–123.0* (0.08–1.24)**
			Air-frying	High-fat foods	61–228.3	No added oil	N/A	3.5–5.0* (0.04–0.05)**	0.04–0.11	9.0–17.4 V sausages: 1.7	22.4–50.4* (0.23–0.51)**

* directly measured VOC concentration or OFP calculation based on directly measured concentration in the chamber (0.1518 m^3).

** estimated VOC concentration or OFP in a real-world kitchen (15 m^3).

(1) Zhang, H.; Wang, X.; Shen, X.; Li, X.; Wu, B.; Li, G.; Bai, H.; Cao, X.; Hao, X.; Zhou, Q.; et al. Chemical characterization of volatile organic compounds (VOCs) emitted from multiple cooking cuisines and purification efficiency assessments. *Journal of Environmental Sciences* **2023**, *130*, 163-173. DOI: 10.1016/j.jes.2022.08.008.
(2) Chen, C.; Zhao, Y.; Zhao, B. Emission Rates of Multiple Air Pollutants Generated from Chinese Residential Cooking. *Environmental Science & Technology* **2018**, *52* (3), 1081-1087. DOI: 10.1021/acs.est.7b05600.
(3) Cheng, S.; Wang, G.; Lang, J.; Wen, W.; Wang, X.; Yao, S. Characterization of volatile organic compounds from different cooking emissions. *Atmospheric Environment* **2016**, *145*, 299-307. DOI: 10.1016/j.atmosenv.2016.09.037.
(4) Kumar, A.; O'Leary, C.; Winkless, R.; Thompson, M.; Davies, H. L.; Shaw, M.; Andrews, S. J.; Carslaw, N.; Dillon, T. J. Fingerprinting the emissions of volatile organic compounds emitted from the cooking of oils, herbs, and spices. *Environmental Science: Processes & Impacts* **2025**, *27* (1), 244-261. DOI: 10.1039/d4em00579a.
